# Single-molecule diffusion and conformational dynamics by spatial integration of temporal fluctuations

**DOI:** 10.1038/ncomms6123

**Published:** 2014-10-06

**Authors:** Maged F. Serag, Maram Abadi, Satoshi Habuchi

**Affiliations:** 1 Biological and Environmental Sciences and Engineering Division, King Abdullah University of Science and Technology (KAUST), Thuwal 23955-6900, Saudi Arabia

## Abstract

Single-molecule localization and tracking has been used to translate spatiotemporal information of individual molecules to map their diffusion behaviours. However, accurate analysis of diffusion behaviours and including other parameters, such as the conformation and size of molecules, remain as limitations to the method. Here, we report a method that addresses the limitations of existing single-molecular localization methods. The method is based on temporal tracking of the cumulative area occupied by molecules. These temporal fluctuations are tied to molecular size, rates of diffusion and conformational changes. By analysing fluorescent nanospheres and double-stranded DNA molecules of different lengths and topological forms, we demonstrate that our cumulative-area method surpasses the conventional single-molecule localization method in terms of the accuracy of determined diffusion coefficients. Furthermore, the cumulative-area method provides conformational relaxation times of structurally flexible chains along with diffusion coefficients, which together are relevant to work in a wide spectrum of scientific fields.

## Abstract (web summary)

Single-molecule localization and tracking technique is widely used to visualize molecular dynamics in life science, yet it fails to detect molecular conformation. Serag *et al*. address this limitation via spatial quantization of temporal fluctuations in the cumulative area occupied by molecules.

## Graphical abstract

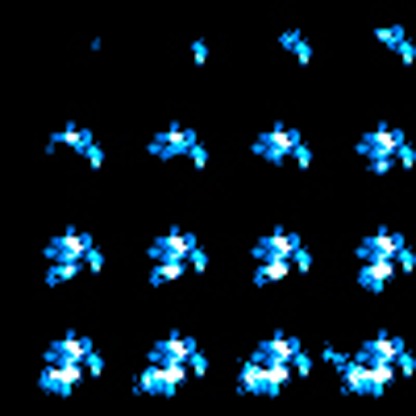

Providing unprecedented insights into the diffusional dynamics of heterogeneous systems in materials and life sciences, single-molecule localization and tracking (SMLT) has been regarded as a powerful tool that offers spatiotemporal resolution not limited by diffraction of light and visualizes the dynamics of individual molecules not masked by ensemble averaging^1^,^2^,^3^,^4^. Notably, SMLT has become a standard technique in the study of subcellular dynamics in live cells, such as analyses of receptors on cell surfaces^5^,^6^, viral infection of cells^7^ and the entire pathway of gene transcription^8^. Furthermore, SMLT has been useful in the study of molecular mechanisms that affect the properties of polymers in materials processing and fabrication^9^. Interestingly, SMLT data can be interpreted to model the standard types of diffusional dynamics, including confined diffusion, random diffusion, directed diffusion and anomalous subdiffusion^10^,^11^,^12^,^13^,^14^,^15^,^16^,^17^,^18^,^19^,^20^.

To analyse the diffusion of single molecules by SMLT, the emitted photon distribution of a fluorescent molecule, at least a few hundred nanometers in size, is fitted by a Gaussian function in both the *x* and *y* dimensions. This fitting aims at highly precise localization of the molecule, typically within a single nanometer to tens of nanometers, by determining the centre of its point-spread function (PSF)^1^,^21^,^22^,^23^. The statistical fit of the ideal Gaussian is repeated with time spacing over a longer period of time, typically a few seconds. The spatiotemporal positions of the molecule are then obtained and spatially connected to generate a temporal diffusion trajectory. The diffusion can be quantitatively characterized from both the spatial and temporal components of the trajectory by calculating the mean square displacement (MSD; Supplementary Fig. 1).

Although the well-established SMLT technology provides accurate spatiotemporal locations of single molecules^24^,^25^, enabling their translational speed to be estimated, it fails to provide essential information about the shape and size of the molecule. Access to shape and size information and integrating it with translational speed measurements would provide important details about molecular diffusion as a crucial life process and reptation as an important physical phenomenon in polymer physics and analytical science. In addition, SMLT introduces several inherent limitations that in practice hinder proper data interpretation. Indeed, SMLT is inefficient and even fails to work in some cases. For example, shape fluctuation of flexible polymer molecules results in large localization error if the molecule is larger than the diffraction-limited size. In the extreme case, the shape of a long molecule temporally fluctuates in space, hampering the accurate localization of its centre-of-mass. Any out-of-focus motion results in large statistical error due to the limited length of the obtained diffusion trajectory. Although recent technologies such as three-dimensional-tracking and electrokinetic trapping offer remarkable performance enhancement of SMLT, these technologies require sophisticated analyses and have low throughput^26^,^27^,^28^,^29^. Statistical and localization errors result in broader distribution of diffusion coefficients of individual molecules. Such errors could affect the interpretation of diffusion data especially in complex analyses such as multimode diffusion studies^30^,^31^. Therefore, there remain many drawbacks to single-molecule localization-based methods that could be overcome by the development of new methods.

Here, we present a method to measure single-molecule diffusion that provides an efficient solution to the limitations described above. Typically, SMLT analyses express molecular motion in terms of accurate spatiotemporal positions of the molecule. Our new method, on the contrary, expresses molecular motion in terms of the increase of the cumulative area occupied by the molecule in space over time, which we call the cumulative-area (CA) method. Through careful adjustment of the number of pixels detected per each time-lapse image, information on translational diffusion, molecular size and frequency of conformational changes can be obtained.

We validate our approach by analysing the statistical distribution of diffusion coefficients of fluorescent nanospheres as calculated by the CA method and the distribution as calculated by SMLT. We further extend the potential usefulness of our approach by analysing the diffusion and conformational dynamics of double-stranded (ds)DNA of different lengths and topological forms, measurements that are critically sensitive to molecular size and conformational changes.

## Results

### Schematic principle of the CA method

**Figure 1:**
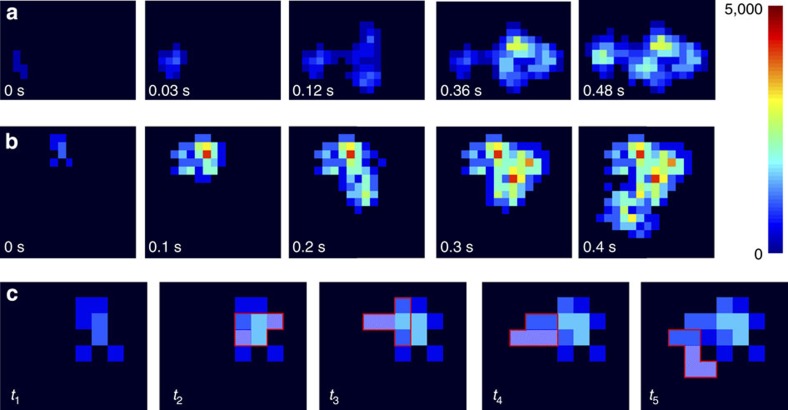
The time-dependent increase in the cumulative area occupied by diffusing molecules. (**a**) The cumulative area occupied by diffusing fluorescent nanospheres and (**b**) YOYO-I labelled linear ColE_1_ DNA gradually increases. Numbers at the bottom indicate time in seconds. The jet colour map represents a.u. values of the cumulative fluorescence intensity of individual pixels. (**c**) Increase in the cumulative area occupied by linear ColE_1_ DNA at time frames *t*_1_ (0 s), *t*_2_ (0.0064, s), *t*_3_ (0.0128, s), *t*_4_ (0.0192, s) and *t*_5_ (0.0256, s) over time. The red-bordered region defines the area occupied by the molecule at each time frame. The dashed pixels represent the area increment at each time frame as a result of random movement of the molecules. Pixel size=0.16 μm.

**Figure 2:**
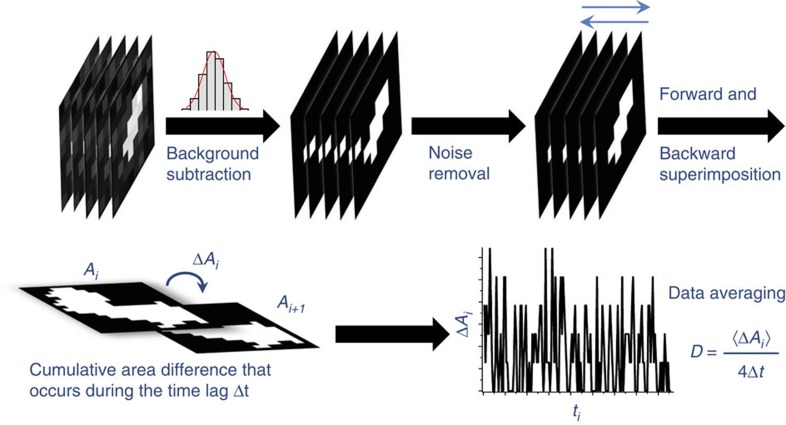
A schematic illustration of the CA method. First, automatic thresholding is done by fitting the frequency distribution of all pixel values in each frame to a Gaussian function. This process is followed by pixel noise removal. Then, the frames are superimposed to generate the cumulative area (*A*_*i*_) at each time frame *t*_*i*_ followed by subtraction to calculate the cumulative-area difference at the time lapse between the adjacent frames (*Δt*). Finally, the cumulative-area difference is used to calculate the diffusion coefficient using the formula *D*=<Δ*A*_*i*_>/4Δ*t*.

The CA approach is based on calculating the increase in the cumulative fluorescent area of moving particles that occurs between time frames *t*_*i*_ and *t*_*i*+1_ (*i*=1,2,…,*n*) (Fig. 1). Given a stack of frames of a time-lapse image sequence of a moving particle, we first remove the background by setting an initial threshold. The threshold is set by fitting the frequency distribution of the intensity of all pixels in each frame with a Gaussian function (Fig. 2, Supplementary Fig. 2). We set the background threshold to *m*+4*s* for the diffusion measurements in which m and s denote the mean and the s.d. of the background intensity distribution determined by the Gaussian fitting. This makes independent background adjustment possible for each frame. The background subtraction is accompanied by removal of noise pixels in all frames. Then, we gradually increase the background threshold to define five pixels (see ‘Validation of the CA analysis on simulated tracks’ in the results section) as the area of space occupied by the molecule in each frame. We note that the number of pixels can be increased to define a complete molecular shape for conformational dynamics analysis (see ‘Methods’ section). The movement of these pixels results in an increase in the area occupied by the molecules across the stack over time (Fig. 1) and can be used to describe the lateral diffusion of the molecule. The quality of the stack is judged based on both the ratio of the number of noise pixels in all frames to the total number of pixels in the stack and the ratio of the number of the empty frames resulting from brief absences of the particle from the field of view to the total number of frames (we call these ratios the internal noise ratio and the dropped frames ratio, respectively; Supplementary Fig. 3). A stack is considered for analysis when the internal noise ratio and the dropped frames ratio do not exceed 20 and 1%, respectively.

To obtain the cumulative area (*M*_*i*_) at each time frame, *t*_*i*_, all frames are superimposed starting at time frame *t*_1_ and continued in sequential order until frame *t*_*i*_. Given the cumulative area throughout the time-lapse image sequence (Fig. 2), the increment of the cumulative area occupied by the molecule that occurs between time frames *t*_*i*_ and *t*_*i*+1_ (Δ*A*_*i*_), because of random molecular motion (Fig. 1), is calculated by the following formula:


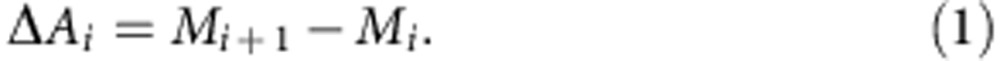


Occasionally, the area difference (Δ*A*_*i*_) is masked by the growing cumulative area. Consequently, the cumulative-area increment could drop to zero (Supplementary Fig. 4). We correct this by reversing the order of the superimposition (see ‘Validation of the CA analysis on simulated tracks’ in the Results section). In such a case, we obtain the cumulative area at time *t*_*i*_ and *t*_*i+*1_ by superimposing all the frames between *t*_*n*_ and *t*_*i*_ and *t*_*n*_ and *t*_*i+*1_. Δ*A*_*i*_ is calculated by the equation,


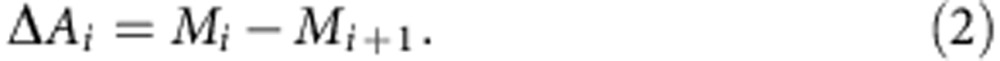


Any zero values in the forward superimposition are replaced with the correct values obtained from the backward superimposition. Finally, the lateral diffusion coefficient (*D*) is obtained from the following formula:


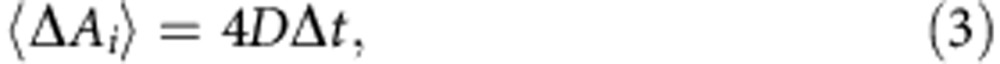


where <Δ*A*_*i*_> is the average area increment at 1Δ*t* and Δ*t* is the time lapse between adjacent frames.

### Diffusion of polymer nanospheres in a buffer

**Figure 3:**
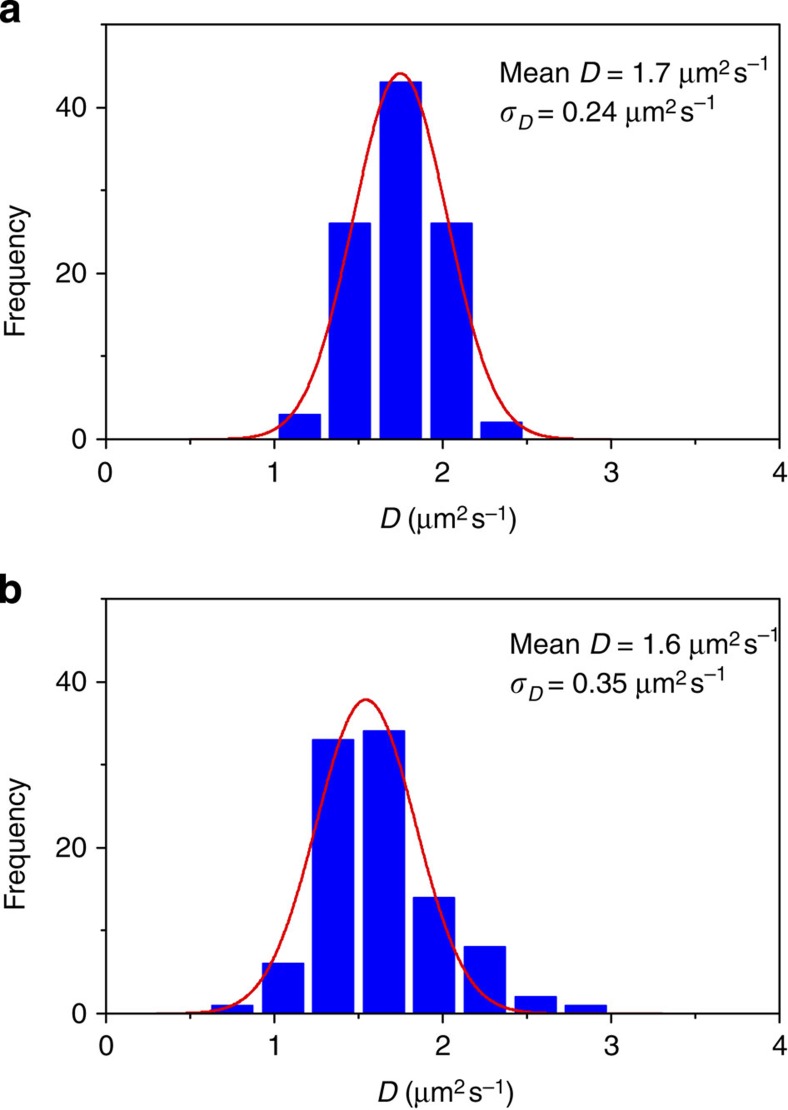
Frequency histograms of the diffusion coefficient determined for nanospheres. The diffusion coefficient values of 0.19 μm fluorescent nanospheres were determined via (**a**) the CA and (**b**) the SMLT-MSD methods. The red lines in **a** and **b** are Gaussian fittings of the frequency distributions.

We validate our approach experimentally by comparing the distribution of diffusion coefficients of approximately 100 cross-linked fluorescent polystyrene nanospheres with a mean diameter of 0.19 μm suspended in 1 mM Tris buffer measured by means of single-molecule fluorescence imaging (see ‘Methods’ section) and analysed by both the CA and SMLT-MSD methods. The expected diffusion coefficient calculated from the Stokes–Einstein equation is 1.74 μm^2^ s^−1^ at 20 °C. Results presented in Fig. 3 show that after fitting the data to the ideal Gaussian, the diffusion coefficient measured by the CA method (1.73±0.24 μm^2^ s^−1^) is in close approximation with that measured by SMLT-MSD (1.63±0.34 μm^2^ s^−1^). Although the distribution appears to be symmetrical around the mean in both analyses, the width of distribution varies to some extent. The s.d. of the diffusion coefficients (*σ*_D_) measured by the CA method (*σ*_D_=0.24) was two-thirds of that measured by the SMLT-MSD method (*σ*_D_=0.35). The distribution of the diffusion coefficient obtained by the SMLT-MSD analysis can be reproduced reasonably by a theoretical statistical probability distribution of diffusion coefficients in a homogeneous environment (see ‘Methods’ section and Supplementary Fig. 5).

The good agreement of the two distributions confirms that the width of the frequency distribution is associated with statistical error in SMLT-MSD. The *σ*_D_ obtained by the CA method (14% of the mean of the *D*) is similar to that obtained by simulation (13–17% of the mean of the *D*, see ‘Simulation of diffusion coefficient of single particles’ section), suggesting that the *D* distribution obtained by the CA method also captures statistical error. Thus, the smaller *σ*_D_ obtained by the CA method as compared with that obtained by SMLT-MSD analysis corresponds to the smaller statistical error in the former method. The smaller statistical error originates from bypassing the localization of the molecule in the CA method

### Simulation of diffusion coefficients of single particles

**Figure 4:**
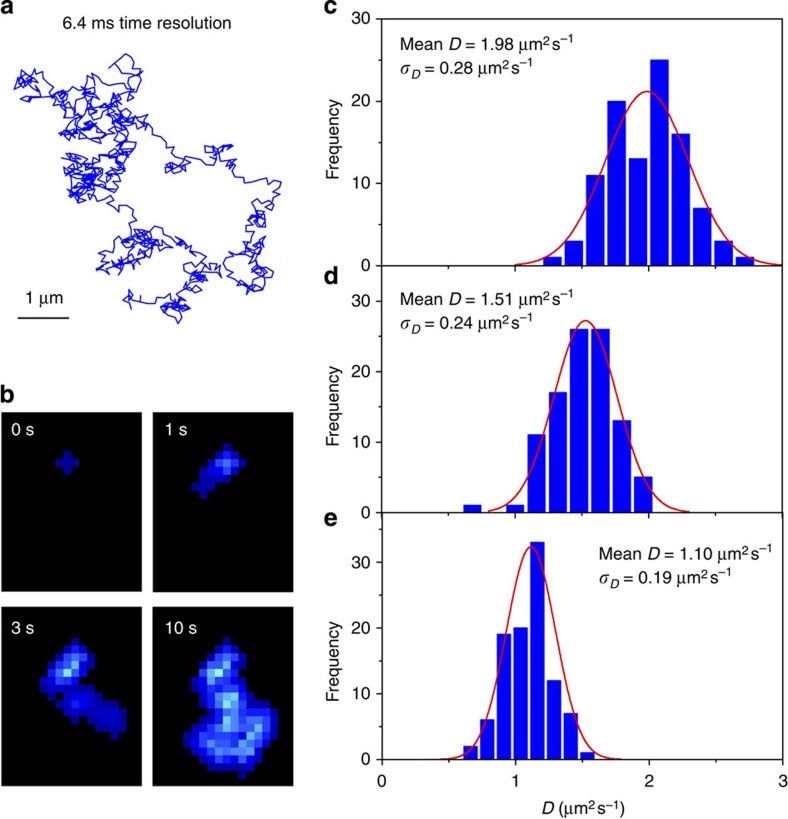
Simulation of the diffusion coefficients of single particles in two dimensions. (**a**) Simulated 2D trajectories of a particle diffusing at *D*=1.0 μm^2^ s^−1^. (**b**) Cumulative areas at time 0, 1, 3 and 10 s calculated using the diffusion trajectory shown in **a**. Frequency histograms of *D* calculated using the simulated trajectories diffusing at (**c**) *D*=2.0 μm^2^ s^−1^, (**d**) *D*=1.5 μm^2^ s^−1^ and (**e**) *D*=1.0 μm^2^ s^−1^ by CA method. The lengths of trajectories in the simulation were set to 50, 100 and 300 frames for particles diffusing at 2.0 μm^2^ s^−1^, 1.5 μm^2^ s^−1^ and 1.0 μm^2^ s^−1^, respectively. The red lines show Gaussian fittings of the histograms.

We evaluate the performance of the CA method using simulated random walk data of single particles. Random diffusion trajectories of 100 particles are generated in two-dimensional (2D) space (see ‘Methods’ section and Fig. 4a). Diffusion trajectories with three different diffusion coefficients (*D*=1.0, 1.5 and 2.0 μm^2^ s^−1^) are simulated and then the *D* values are calculated using the CA method (Fig. 4b). Note that the *D* values and trajectory lengths in the simulation lie within the range of experimentally obtained diffusion coefficients and the trajectory lengths of the DNA molecules used in this study. The frame rate (156 frames s^−1^) is also set at the value for the single-molecule imaging experiments on DNA molecules. Good agreement between the simulated diffusion values (1.0, 1.5 and 2.0 μm^2^ s^−1^) and the calculated values (1.10, 1.51 and 1.98 μm^2^ s^−1^) is obtained, demonstrating the accuracy of the CA method (Fig. 4c–e). Much smaller diffusion coefficients can also be reliably calculated provided that a suitable sampling rate is chosen (Supplementary Fig. 6). The results of the simulation shows that the sampling rate should be associated with a large-enough displacement to result in a detectable area increment over time. This ensures that the short displacements that could be generated at fast sampling rates are not masked by the area occupied by the molecule in each frame. A similar prerequisite is crucial in SMLT-MSD analysis where the sampling rate should result in a displacement that is much larger than the localization accuracy obtained after the 2D Gaussian fitting.

### Diffusional dynamics of dsDNA

Owing to ease, reliability and precise control of its synthesis, DNA is well established as a model biopolymer for understanding fundamental phenomena in polymer physics^32^. Therefore, studying the diffusional dynamics of single DNA molecules is a useful endeavour to determine the diffusion behaviour of isolated polymers in infinite dilutions.

**Figure 5:**
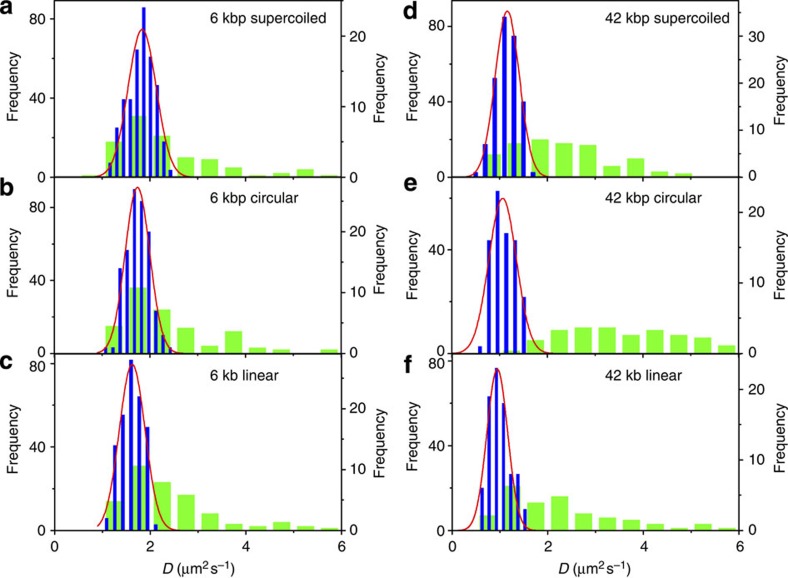
Frequency histograms of the diffusion coefficients determined for DNA. Different topological forms of (**a**–**c**) ColE_1_ (6 kbp) and (**d**–**f**) Charomid (42 kbp) DNA are analysed and the diffusion coefficient values are determined via the cumualative area (blue bars) and the SMLT-MSD (green bars) methods for supercoiled (**a**,**d**), relaxed circular (**b**,**e**) and linear (**c**,**f**) forms. The red lines are Gaussian fittings of the respective frequency distributions.

To provide further evidence of the reliability and validity of our method, we measure the distribution of diffusion coefficients of YOYO-I labelled ColE_1_ (6 kbp) and Charomid (42 kbp) dsDNA of different topologies (Table 1 and Fig. 5). The peaks of the *D* distribution of supercoiled and relaxed circular ColE_1_ DNA measured by the CA method are in close approximation with values obtained by SMLT-MSD analyses (Fig. 5a,b). A slight deviation in the peak is observed in the *D* distributions of the linear form of ColE_1_ DNA obtained by both methods (Fig. 5c). In contrast to the analogous peak values obtained by the two methods, a marked difference in the width of the distribution is observed. The widths of the *D* distributions obtained by the CA method (*σ*_D_ is approximately 15% of the mean of the *D*) are similar to those obtained using fluorescent nanospheres and a simulated diffusion trajectory (Table 1). On the other hand, much broader distributions (*σ*_D_ is approximately 40–65% of the mean of the *D*) are obtained by the SMLT-MSD method (Table 1). The distributions are indeed much broader than the theoretically calculated statistical probability distribution of diffusion coefficients in a homogeneous environment (see ‘Methods’ section and Supplementary Fig. 7). The calculated radii of gyration (*R*_G_) of supercoiled, relaxed circular and linear ColE_1_ DNA are 0.1, 0.13 and 0.21 μm, respectively^33^,^34^. Although the *R*_G_ of ColE_1_ DNA in different topological forms are comparable to the width of the PSF of our experimental setup, the broadening of the *D* distribution obtained by the SMLT-MSD analyses indicates that the conformational changes even at this length scale affect the analysis of the diffusion behaviour in the SMLT-MSD method. The absence of the broadening of the *D* distribution in the CA analysis highlights the advantage of the CA method over the SMLT-MSD analysis.

**Figure 6:**
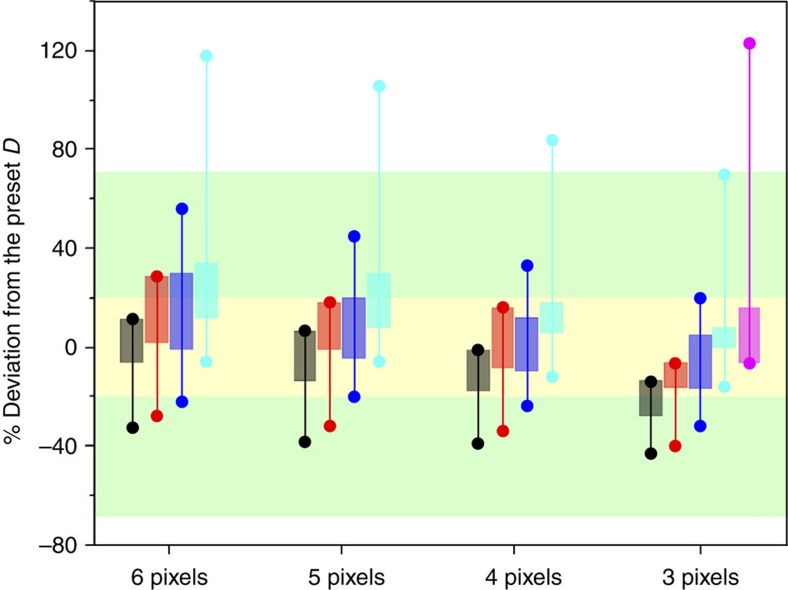
Accuracy of the calculated diffusion coefficients. Random diffusion trajectories with the pixel size of 160 nm are generated and analysed by the CA method. The green zone represents the statistical error (±68%) of the diffusion coefficient calculated by the SMLT-MSD analysis of 100 particles. The yellow zone represents the threshold range (±20%) we set to validate the calculated diffusion coefficients. The values between the two circles on the same line represents the range of the calculated diffusion coefficients obtained for the simulated trajectories with preset diffusion coefficients of 2.0 (black), 1.5 (red), 1.0 (blue), 0.5 (cyan) and 0.2 μm^2^ s^−1^ (magenta) by varying the tracking time from 0.32 to 32 s (Supplementary Table 1). The percentage deviations of the calculated means obtained within the range of the experimental tracking time (see Supplementary Table 3) are plotted as bars for each preset diffusion coefficient.

The difference between the *D* distributions calculated by both methods is more remarkable for the much longer 42 kbp Charomid DNA (*R*_G_≈0.65 μm (refs 35, 36), Fig. 5d–f). This is associated with significant broadening of the distributions measured via SMLT-MSD and inconsistent peak values. Since the *R*_G_ of Charomid DNA is much larger than the PSF of the microscope, the fluctuations in conformational changes of the DNA are evident within each trajectory (Fig. 6a). This hampers accurate determination of the centre-of-mass of the molecules. Furthermore, the anisotropic shape of the fluorescently labelled Charomid molecules introduces considerable errors in the 2D Gaussian fitting. This, in turn, makes the tracking process fail quite quickly because of large fitting outliers. Although several algorithms have been successfully applied to optimize the tracking conditions of small and elongated molecules^37^,^38^,^39^, these methods rely on the fitting of the image with a 2D function that limit their applicability to conformationally unstable molecules. The CA method circumvents the localization algorithm and shows narrower and much better symmetrical distribution around the mean diffusion coefficient (Fig. 5, Supplementary Fig. 8). Indeed, the widths of the *D* distribution obtained by the CA analysis (*σ*_D_ is approximately 20 % of the mean of the *D*) are comparable to those obtained for the nanospheres and shorter 6 kbp ColE_1_ DNA. Furthermore, the mean *D* obtained by the CA analysis (0.99 μm^2^ s^−1^ for the linear form of 42 kbp Charomid DNA) is comparable to the ensemble-averaged *D* obtained for a similar length of DNA (0.80 μm^2^ s^−1^ for the 48.5 kbp λ DNA)^40^. These results demonstrate the robustness of the CA method and its applicability to a wide range of the samples including large, conformationally unstable molecules.

### Validation of the CA analysis on simulated tracks

We validate the input parameters of the CA method using simulated data to benchmark its performance to provide reliable estimates of the diffusion coefficients. First, we calculate the diffusion coefficients in the absence of the backward superimposition correction at different tracking times (0.32 s ~32 s; frame rate=6.4 ms; Supplementary Fig. 9). We found that the calculations underestimate the simulated diffusion coefficients (Supplementary Fig. 9c) because the growing area masks the cumulative-area increment (Δ*A*_*i*_=0). As the backward superimposition correction is applied, reliable estimates of diffusion coefficients are obtained (Supplementary Fig. 9c). The observed gradual decrease of the calculated diffusion coefficients by increasing the tracking time (Supplementary Fig. 9c) is attributed to an increasing probability that the masked area (Δ*A*_*i*_=0) could coincide in both the forward and the backward superimpositions as the tracking time is increased. We calculate the mean diffusion coefficients (*D*_μ_) of 100 simulated molecules after varying three parameters to evaluate the validity of the CA method (Supplementary Tables 1 and 2): (1) the number of pixels used to represent the area occupied by the molecule in space (three to six pixels), (2) the preset displacements (*d*=50–240 nm corresponding to 0.3–1.5 × of the pixel size and corresponding to preset diffusion coefficients (*D*_p_)=0.1–2.25 μm^2^ s^−1^ at frame rate=6.4 ms) and (3) the tracking time (0.32 to 32 s corresponding to the typical tracking times for the molecules diffusing at the preset displacements). To judge the reliability of the calculated *D*_μ_ for each of the parameters, we set an accuracy threshold range, equal to ±20% of the *D*_p_ (Fig. 6 and Supplementary Tables 1 and 2). When the *D*_μ_ values are outside of this range, we consider them as unreliably estimated values. It is important to note that statistical errors of ±68% (±3.29 s) of the calculated mean are obtained by the SMLT-MSD analysis for 100 particles (Fig. 3b). Therefore, the range of the accuracy threshold (±20%) represents only 30% of the statistical errors (Fig. 6). Our simulations predict that the calculated *D*_μ_ values with the systematic uncertainty originating from the probabilistic coincidence of the masked area (Δ*A*_*i*_=0) in both superimpositions lie within the ±20% threshold limit for the simulated particles. This explains the narrow distribution of the diffusion coefficients obtained by the CA method. The simulations further indicates that accurate calculations requires a trade-off between pixel size and the preset displacement. According to the simulations, the diffusion coefficient of a particle can be reliably estimated using the CA method by representing the particle with three to five pixels and within the range of displacements equals to 0.55–1.4 × of 160 nm pixel size. Shorter displacements (88–50 nm), can be accurately measured by the CA method using pixel size of 100 nm (Supplementary Table 2). It is important to underline that a wide range of diffusion coefficients can be analysed by choosing an appropriate frame rate, similar to SMLT-MSD.

**Figure 7:**
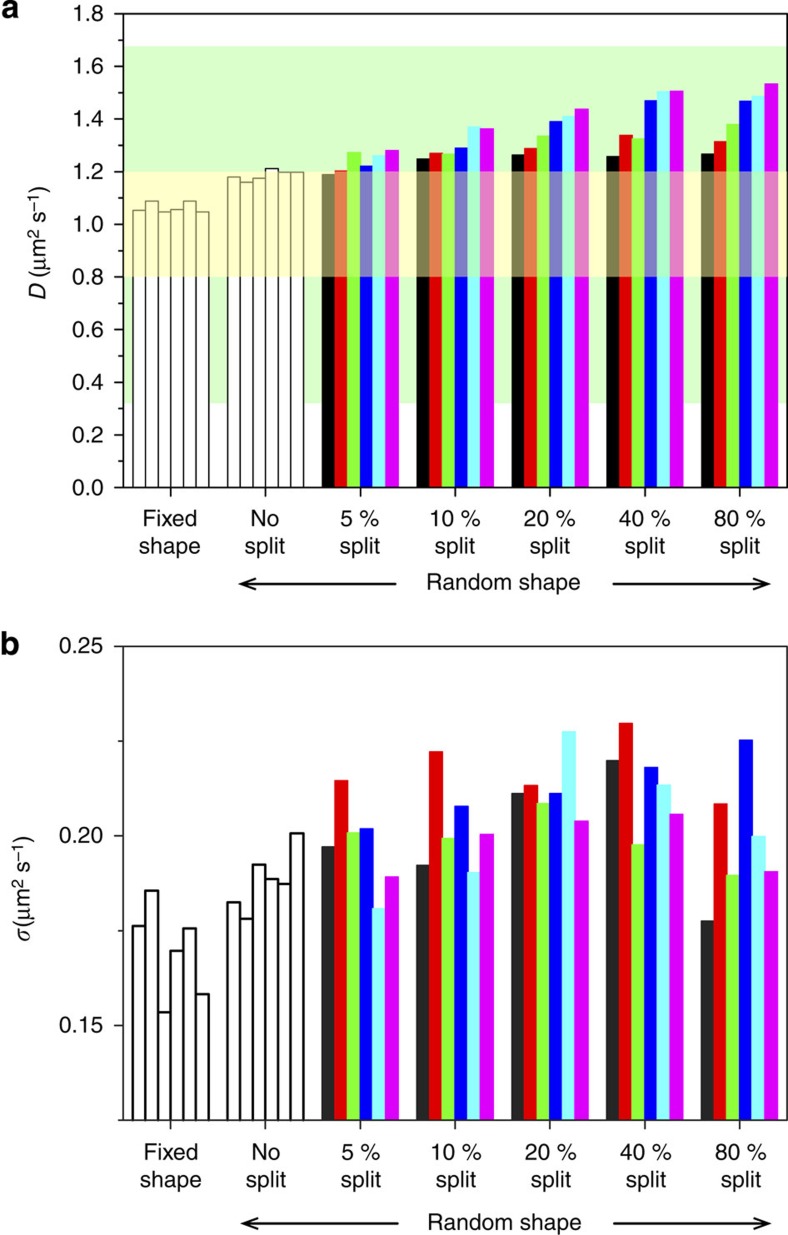
Effect of the fluctuations of the molecular shape on the calculated diffusion coefficients. 2D trajectories of five pixels diffusing at 1 μm^2^ s^−1^ are simulated (1,000 tracks, each consists of 500 steps with 0.0064, s time resolutions). The diffusion coefficients are calculated using the CA method. (**a**) Calculated diffusion coefficients of the simulated tracks using: (1) five pixels of fixed shape (the six columns show results of six runs of the simulation), (2) five pixels of random shape without splitting (the six columns show results of six runs of the simulation) and (3) five pixels of random shape with 5 (25 frames), 10 (50 frames), 20 (100 frames), 40 (200 frames), 80% (400 frames) of the total frames showing splitting. The colour code illustrates the split distance in pixels in both *x* and *y* directions: 2 (black), 3 (red), 4 (green), 5 (blue), 6 (cyan) and 7 pixels (magenta). The green zone represents the s.e. (±68%) of the diffusion coefficient calculated by the SMLT-MSD analysis of 100 particles. The yellow zone represents the threshold range (±20%) we set to validate the calculated diffusion coefficients. (**b**) s.d. calculated for the diffusion coefficients in **a**.

We further investigate the effect of the shape fluctuation of the molecule on the CA analysis. In the CA analysis, the area occupied by the molecule is described by five brightest pixels in the image after the removal of noise pixels (Supplementary Fig. 3). The floppy nature of DNA causes the continuous fluctuation of the shape of the five pixels. Indeed, the shape of the five pixels obtained for the DNA molecule (Supplementary Fig. 10b) shows more fluctuations as compared with that obtained for the nanospheres (Supplementary Fig. 10a). Furthermore, the DNA molecule occasionally shows splitting of the five pixels into two parts due to the slight defocusing of the molecule (Supplementary Fig. 10b). To study the effects, we generated simulated diffusion trajectories with randomly varied shapes of the five pixels (Supplementary Fig. 11a). The splitting of the pixels at variable splitting distances is further introduced to the simulated trajectories in varying proportions (Supplementary Fig. 11b). The CA analysis on the simulated trajectories (Fig. 7 and Supplementary Fig. 12) showed that the calculated diffusion coefficients were located within the 20% accuracy threshold we set to judge the validity of the method (Fig. 7a). The result demonstrates that the CA analysis tolerates the shape fluctuations of the five pixels.

While the diffusion coefficient is slightly overestimated when the splitting of the five pixels occurs (Fig. 7a), the CA analysis is valid as long as the splitting is smaller than three pixels and the proportion of the split image is less than 5% of the total frames (that is, the diffusion coefficient is within the 20% accuracy threshold, Fig. 7a). Since the splitting of the pixels is related to the defocusing of the image, this result demonstrates that the CA analysis tolerates the slight defocusing. In addition, the s.d. of the diffusion coefficients calculated from 1,000 simulated trajectories remain almost constant regardless the splitting distance and the proportion of the split image (Fig. 7b). This reinforces the special performance of the CA method in analysing the diffusion coefficient of conformationally unstable molecules as compared with SMLT-MSD.

**Figure 8:**
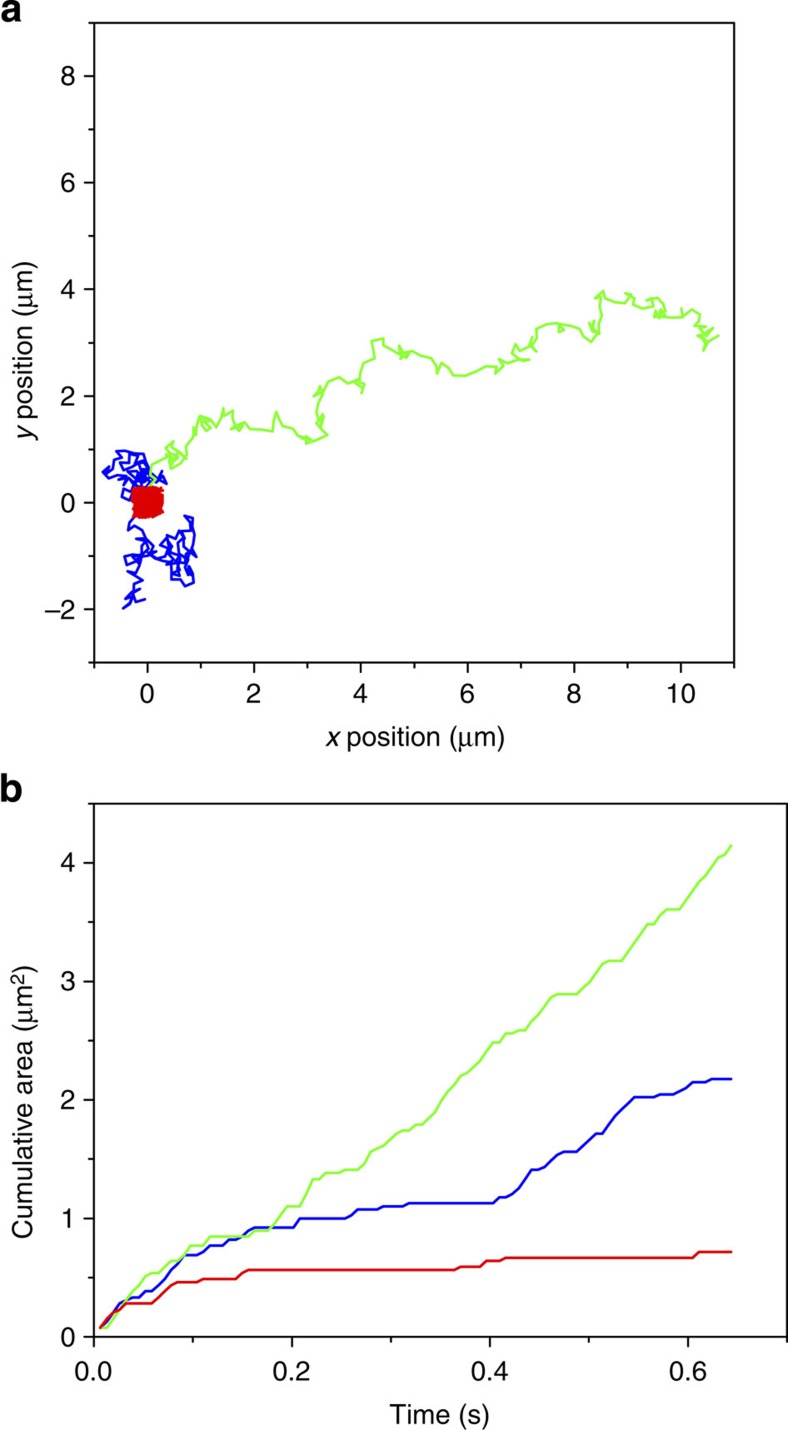
Simulation of different diffusion modes of single particles. (**a**) Simulated 2D trajectories of a particle diffusing in a random (blue), directed (green) and confined fashion (red) at *D*=1.0 μm^2^ s^−1^ (frame rate=6.4 ms). (**b**) Cumulative areas at time 0–0.64 s calculated using the trajectories of random (blue), directed (green) and confined diffusion (red) displayed in **a**. Enlarged view of **a** is shown in Supplementary Fig. 13.

Random, directed and confined diffusion can be probed with the CA method in similar way to MSD analyses (Fig. 8 and Supplementary Fig. 13). Since particles, moving in a pure Brownian fashion, occasionally revisit the pre-explored areas of space, the overall cumulative area resulting from the sequential superimposition between time frames *t*_1_ and *t*_*n*_ tends to show irregular, non-linearly increasing cumulative pattern (Fig. 8b, blue line). When the particles show a directed motion, a linear increase is observed because the particles tend to move away from its original location rather than re-exploring the space (Fig. 8b, red line). When the diffusion is restricted to a confined space, the cumulative area rapidly reaches an asymptote where further area increase is constrained (Fig. 8b, green line). Altogether, these results demonstrate the versatile applicability of the CA method in probing random as well as anomalous diffusion.

### The conformational dynamics of Charomid DNA

Diffusion of a long and flexible chain (for example, Charomid DNA) is believed to be coupled with its conformational relaxation dynamics. However, simultaneous detection of these two parameters has been challenging due to the lack of suitable methods. One of the advantages of using the CA method over other methods is that it can provide direct information about conformational changes in parallel with diffusion dynamics.

**Figure 9:**
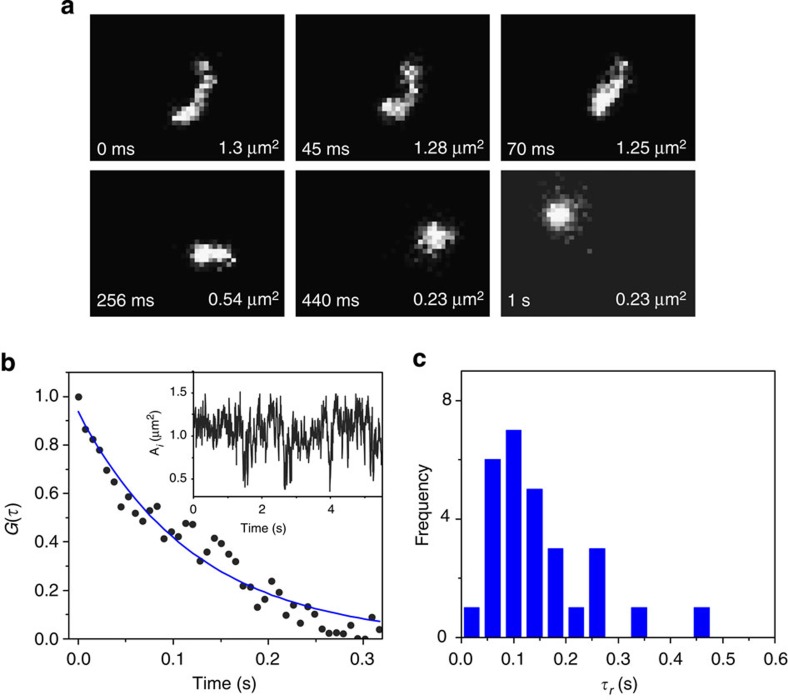
Conformational dynamics of Charomid DNA. (**a**) Fluorescence images of single DNA molecules (42 kbp) at different time frames. The molecular area in μm^2^ is illustrated for each image. (**b**) An autocorrelation plot of the area occupied by a single Charomid DNA. The blue line shows a fitting with a single-exponential decaying function. (Inset) Time lapse of the area occupied by a single Charomid DNA (42 kbp) molecule. (**c**) Frequency histogram of the calculated relaxation time.

The spontaneous conformational relaxation of spatially isolated DNA can yield dramatic change in the area occupied by the molecule in space (Fig. 9a). To measure the timescale of this change, the area occupied by the molecule (*A*_*i*_) at a given time, *t*_*i*_, is calculated (Fig. 9b, inset). The relaxation time of the molecule (*τ*_R_) is estimated by the autocorrelation analysis of *A*_*i*_ using the following formula:


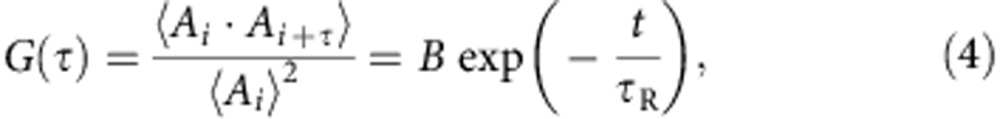


where *τ* denotes the time lag. The autocorrelation plot obtained for the linear form of Charomid DNA molecules (42 kbp) with a 1-s sampling time can be fitted well with a single-exponential decay function (Fig. 9b). The average correlation time of 144 ms was obtained (Fig. 9c), which is in close approximation with the reported relaxation time (*τ*_R_≈200 ms) of λ DNA (48.5 kbp) (ref. 40). The relaxation time of the cyclic form of the Charomid DNA calculated in the same way (*τ*_R_≈80 ms) is shorter than that of its linear counterpart, consistent with the topology-dependent relaxation time obtained in simulation studies^41^,^42^. These results imply a direct connection between the relaxation time obtained by the CA method and the conformational relaxation of the chains.

The anti-Brownian electrokinetic trap method and the CA method produce similar results^35^. In the anti-Brownian method, however, the diffusion coefficient is deduced from the applied force on the molecule. This makes the analysis cumbersome and complicated, especially when the diffusion is complex and inhomogeneous^31^. In contrast, the CA method simultaneously reports the diffusion coefficient and the relaxation time of the molecule in a straightforward way (Supplementary Figs 14 and 15) in the absence of external forces that might affect the conformational state of the trapped molecule. Combining reliable estimates of relaxation times with accurate diffusion measurements suggests a new way to investigate the heterogeneous dynamics of polymers in a crowded environment.

## Discussion

The method we present here circumvents molecular localization, the cornerstone of single-molecule diffusion measurements and overcomes its inherent limitations. We validate the method using fluorescent nanospheres and dsDNA molecules of different lengths and topological forms. We found that the performance of the CA method surpasses the SMLT method in terms of reproducible measurement of relaxation times and diffusion dynamics with a narrow distribution of the values obtained for a particular DNA size and topological form.

Our new method holds promise to advance single-molecule fluorescence microscopy studies, which are relevant to a wide spectrum of scientific fields. From fundamental physics to materials science to chemistry, biology and analytical science, translational diffusion and conformational dynamics are, indeed, central to multidisciplinary studies of polymers. In closing, we offer suggestions on the immediate applicability of our method in these divergent fields.

In chemistry, physics and biological science, there is considerable fundamental interest in the effect of molecular topology on entangled biopolymer dynamics. In particular, our method can be reliably used to unveil nanoscale heterogeneity and molecular dynamics in complex systems. Our method, for example, can provide insights into anomalous diffusion including directional flow and constraint diffusion (see Fig. 8). In the field of soft condensed matter physics and gel electrophoresis, the flow behaviour of entangled polymers, viscous and elastic deformation and orientation in electrophoretic flow and the effect of molecular topology on electrophoretic mobility have rapidly become foci of research activity. We conceive of no better strategy to track such behaviours than by using the CA method, which promises significant analytical improvement over other methods. In polymer physics and materials science, understanding the diffusional dynamics of polymers is the key to characterizing the rheological properties of synthetic polymers and nanocomposites. Indeed, the viscoelastic properties of these materials can be discerned after diffusional and conformational change measurements using the CA method. Work in this area is motivated by the need to develop new materials that are relevant to industrial products, including plastics, fibres, emulsifiers and countless others. Additional methodological developments, including three-dimensional area tacking and multicolour imaging will broaden the horizon of applications of the CA method. Finally, we believe that our method will work in conjunction with super-resolution microscopy to unravel the dynamics of complex biosystems that continue to remain beyond experimental realization.

## Methods

### Fluorescent materials

Suncoast yellow fluorescent polymer nanospheres (excitation/emission maxima 540/600 nm) of nominal size (0.19 μm; 2.653 × 10^12^ nanspheres ml^−1^) were purchased from Bangs Laboratories, Inc. (Fishers, IN, USA). The nanospheres were diluted with 10 mM TRIS buffer (pH 8) to a concentration of 1.5 × 10^6^.

ColE_1_ (6 kbp) and Charomid (42 kbp) DNA were purchased from Nippon Gene (Tokyo, Japan). The restriction enzyme SmaI (New England Biolabs (UK)) was used to prepare the linear form, and Topoisomerase I (New England Biolabs) was used to prepare the relaxed form. The DNA molecules were labelled with YOYO-I (Molecular Probes, Carlsbad, CA) (dye molecules to base pair ratio of 1:5) as described in ref. 32.

### Single-molecule fluorescence imaging setup

The single-molecule imaging experiments were carried out on a custom-built widefield epifluorescence microscope setup. Two solid-state lasers were used for illumination: a CW 60 mW 488 nm (MLD, Cobolt, Solna, Sweden) and a CW 100 mW 532 nm (Samba, Cobolt). In this setup, the laser beams pass through an acousto-optic tunable filter (AOTF; AA Optoelectronic, Orsay, France), which allows the intensity of the individual laser lines to be independently controlled. The AOTF is synchronized to an iXon3 897 EMCCD camera (Andor Technology, Belfast, Ireland) to illuminate the sample only during the light-integrating phase and thus to reduce photobleaching. The collimated laser beams are directed through a × 5 beam expander (Thorlab, Newton, NJ, USA), which in combination with an achromat lens in front of the microscope entrance provides widefield Kohler illumination of the sample. The fluorescence images were recorded using an inverted IX71 microscope (Olympus, Japan) with an Olympus Plan Apochromat × 100 NA 1.49 oil immersion objective lens. The fluorescence light coming from the sample was collected again by the objective lens and sent through the side port of the microscope towards the EMCCD camera. The fluorescence light is separated from the laser excitation light using a dichroic mirror (FF506-Di03) and an emission filter (FF01-609/181) obtained from Semrock (Lake Forest, IL, USA). The fluorescence images were recorded for 6.4 ms sampling time with a pixel size of 160 nm. The image acquisition was done using the Andor iQ imaging software. Approximately, 100 single-molecule diffusion trajectories were analysed in each experiment.

### Single-molecule fluorescence image analysis

Analyses of the diffusion trajectories by the area-based method and SMLT-MSD method were performed off-line using a routine written in Matlab. In the SMLT-MSD analysis, the positions of the molecules in each image were determined by using two-dimensional Gaussian fitting:


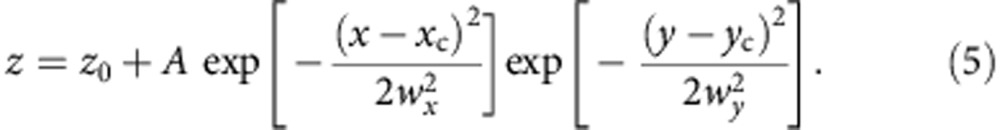


The MSD was calculated by using the following formula:


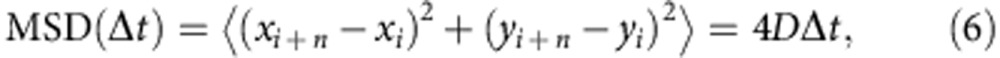


where *x*_*i*+*n*_ and *y*_*i*+*n*_ describe the molecular spatial position following a time interval, Δ*t*, given by *n* frame rate after starting at positions *x*_*i*_ and *y*_*i*_. *D* is the lateral diffusion coefficient and is calculated from the slope of the MSD plot (Supplementary Fig. 1).

### Probability distribution of diffusion coefficients

The statistical probability distribution of the diffusion coefficient in a homogeneous environment is described by,


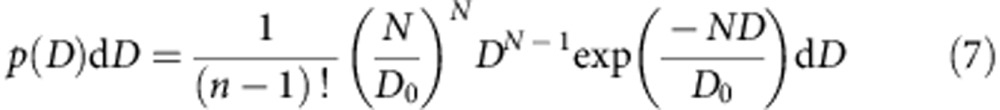


where *N* is the number of independent pairs (number of displacements), *D*_0_ is the true mean diffusion coefficient and *D* is the diffusion coefficient for an individual trajectory.

### Simulation of the random-walk of single molecules

The random-walk trajectory was constructed using a routine written in Matlab starting at (*x*,*y*)=(0,0). The random displacements were generated using a distribution function (*q*) expected from the normal diffusion theory:


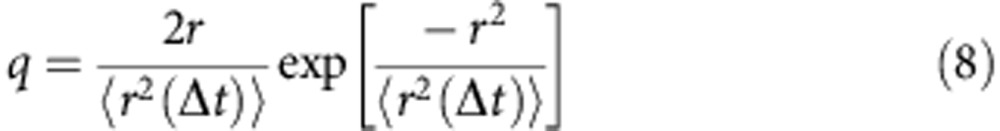


where *r* and Δ*t* denote the displacements and the time lag. Thus, ‹*r*^2^(Δ*t*)› corresponds to the MSD at the time lag Δ*t*. The angles between two successive displacements were generated based on random values between 0 to 360°. The obtained (*x*,*y*) positions were then used to define the centre of the same number of pixels (five pixels) used in our experiments. The frequency distribution calculated for 100 simulations is shown in Fig. 4.

### Conformational dynamics of DNA

To measure the relaxation time of single DNA molecules, the molecular area was defined by specifying an upper threshold of 40–60 pixels. The upper threshold was determined based on the largest number of pixels obtained after background subtraction in each video recording. The largest number of pixels in each recording represents the fully extended DNA molecules. The area fluctuations of approximately 100 Charomid DNA molecules (42 kbp, 0.1 mg ml^−1^) were autocorrelated and the autocorrelation plot was fitted to a single-exponential decay using Origin (originlab Version 9.0).

## Author contributions

M.F.S. introduced the concept, designed the simulations and the analytical approach, conducted the experiments, wrote the Matlab scripts, analysed the data and wrote the manuscript. M.A. assisted on the experiments and data analysis. S.H. supervised the study and wrote the manuscript.

## Additional information

**How to cite this article:** Serag, M. F. *et al*. Single-molecule diffusion and conformational dynamics by spatial integration of temporal fluctuations. *Nat. Commun.* 5:5123 doi: 10.1038/ncomms6123 (2014).

## Supplementary Material

Supplementary Information Supplementary Figures 1-15 and Supplementary Tables 1-3

## Figures and Tables

**Table 1:** Measured diffusion coefficients for DNA molecules using the cumulative-area and SMLT-MSD methods.

**DNA construct**	**Diffusion coefficients (mean±s.d.), μm**^**2**^** s**^−1^*					
	**Supercoiled**		**Circular**		**Linear**	
	**CA**	**SMLT**	**CA**	**SMLT**	**CA**	**SMLT**
ColE_1_ (6 kbp)	1.80±0.30	2.62±1.72	1.74±0.24	2.52±1.32	1.61±0.25	2.38±0.97
Charomid (42 kbp)	1.14±0.24	2.41±1.20	1.10±0.24	4.19±1.81	0.99±0.22	2.30±1.25

CA, cumulative area; MSD, mean square displacement; SMLT, single-molecule localization and tracking.

^*^Diffusion coefficients were determined based on calculating the arithmetic mean of values in Fig. 5.
