# Supplementary material for: Single-molecule diffusion and conformational dynamics by spatial integration of temporal fluctuations
Source: Nat Commun. 2014 Oct 6;5:5123. doi: 10.1038/ncomms6123 (PMC4205855; doi:10.1038/ncomms6123)
Supplement: Supplementary Information — Supplementary Figures 1-15 and Supplementary Tables 1-3 [file ncomms6123-s1.pdf]

Supplementary figures

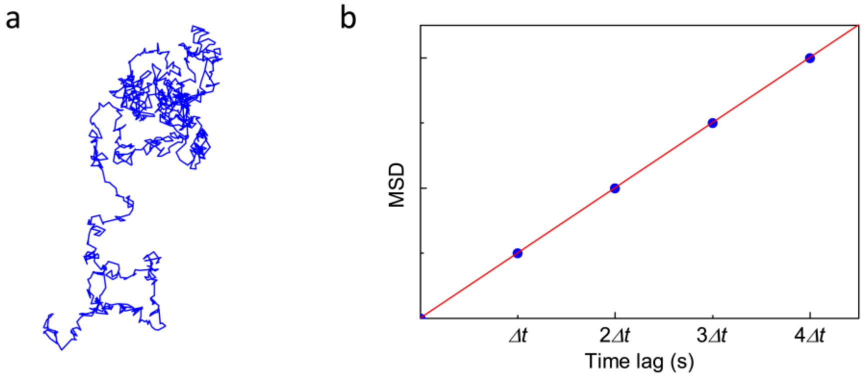

Supplementary Figure 1. Single molecule diffusion analysis using the SMLT-MSD method. Spatiotemporal locations of a single molecule were determined by fitting the fluorescence images with a 2D-Gaussian function. (a) The diffusion trajectory was obtained by connecting the spatiotemporal positions of the molecule. (b) MSD-Δt plot for random diffusion. The diffusion coefficient (D) can be calculated from the slope of the plot using the formula, MSD(Δt) = $4D\Delta t$.

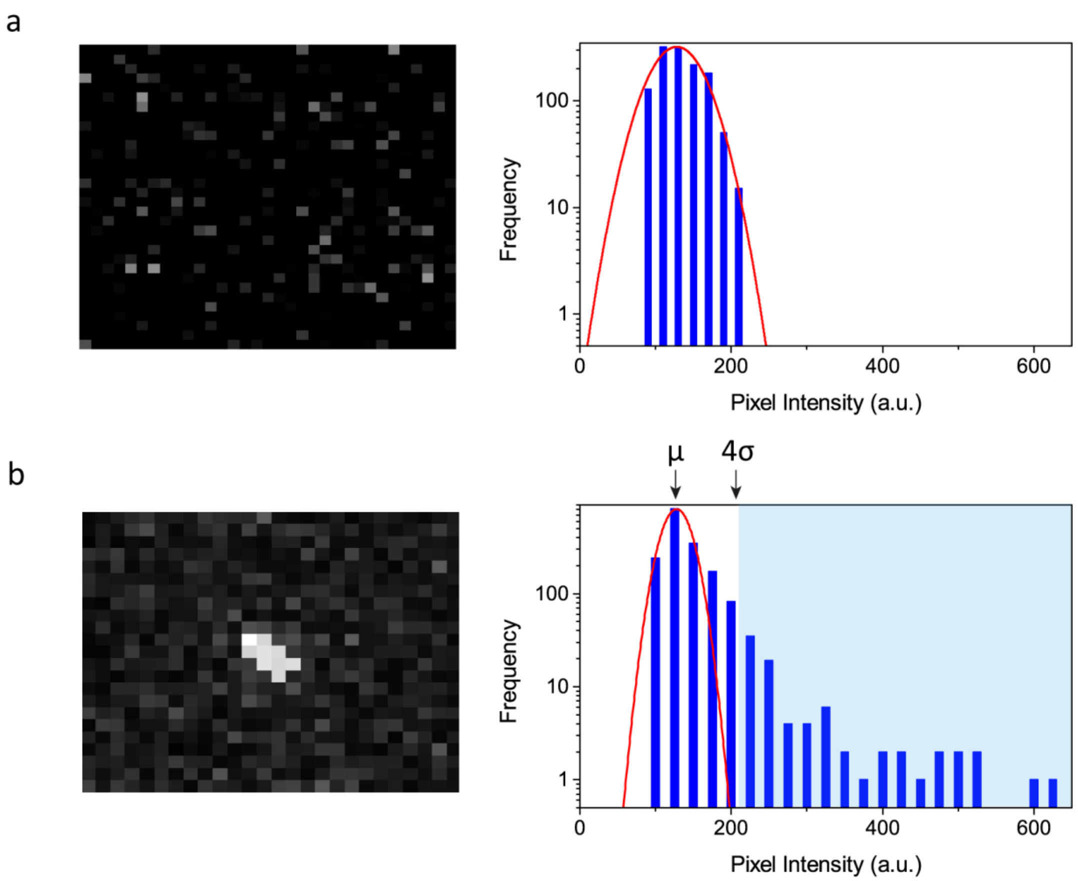

Supplementary Figure 2. Automatic background thresholding of single-molecule images. (a) An epifluorescence image of blank Tris buffer (left) and a corresponding frequency distribution of the pixel intensity (right, blue bars). The red line represents the Gaussian fitting to the distribution. (b) An epifluorescence image of YOYO-I labeled linear $ColE_1$ DNA in Tris buffer (left) and a corresponding frequency distribution of the pixel intensity (right, blue bars). The background threshold was automatically selected by specifying the mean (μ) and the standard deviation (σ) after Gaussian fitting (right, red lines).

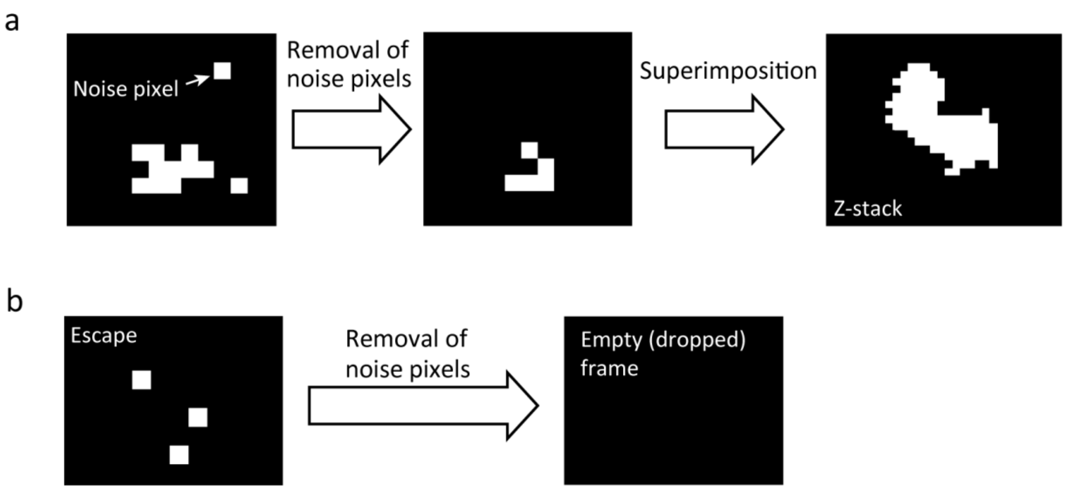

Supplementary Figure 3. Removal of individual noise pixels. After background subtraction, all frames are converted to binary images. (a) The noise pixels and defocused parts of the molecule are removed, leaving a limited number of pixels in each frame. The superimposed frames (referred to as Z-stack) at time t_i (i = 1, 2, … , n) is used to calculate the molecular diffusion. (b) Dropped frames resulting from transient absence of the molecule from the field of view (should not exceed 1 % of the total frames).

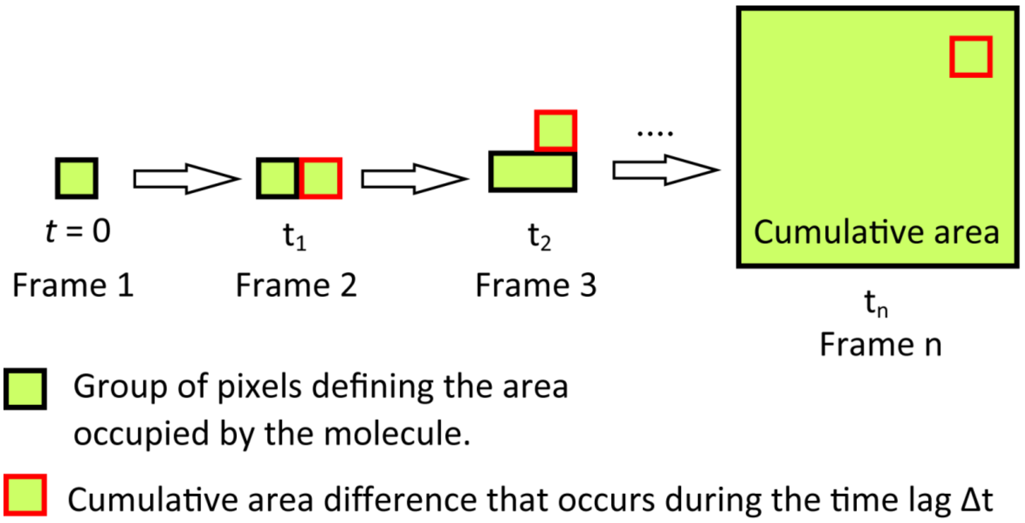

Supplementary Figure 4. As the cumulative area increases in size, it is possible that the particle movement (red bordered squares) becomes masked by the growing cumulative area (the big black bordered square).This is resolved by reversing the order of the superimposition of the frames.

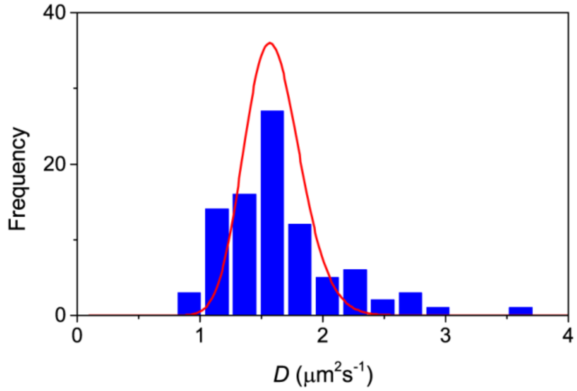

Supplementary Figure 5. Frequency histogram of the diffusion coefficients determined by the SMLT-MSD analyses for 90 trajectories of equal length (50 frames). The red line shows a calculated theoretical statistical distribution corresponding to the diffusion of single molecules in a homogeneous environment, with the diffusion coefficient given by the mode of the frequency histogram (Eq. 7).

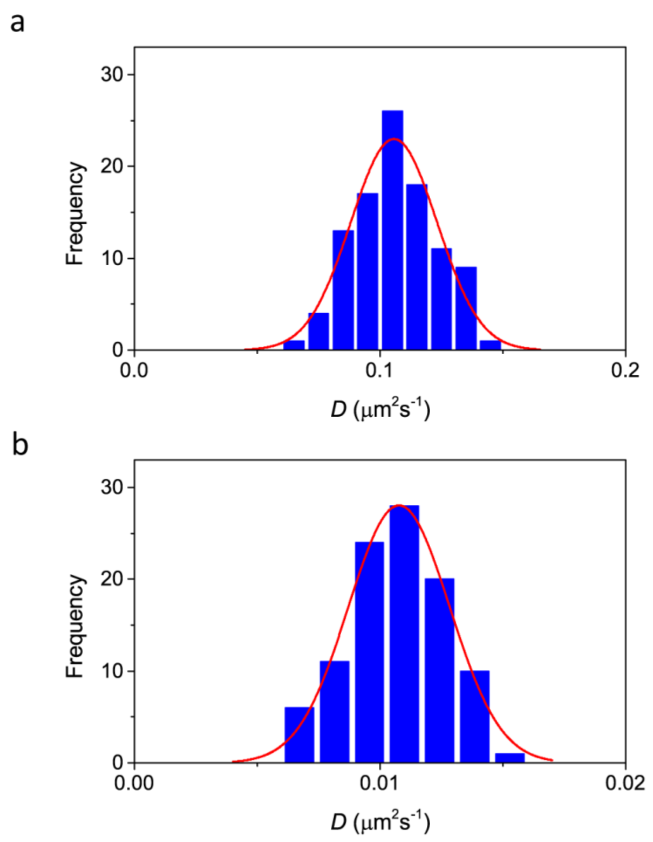

Supplementary Figure 6. Simulation of the diffusion coefficient of single particles in two dimensions. 2D trajectories of a particle diffusing at 0.1 $\mu m^2 s^{-1}$ and 0.01 $\mu m^2 s^{-1}$ were simulated (1000 steps with 0.064 s and 0.64 s time resolutions). The diffusion coefficients were calculated using the cumulative-area method and plotted in frequency histograms: (a) 0.1 $\mu m^2 s^{-1}$ and (b) 0.01 $\mu m^2 s^{-1}$. The red lines show Gaussian fittings of the histograms.

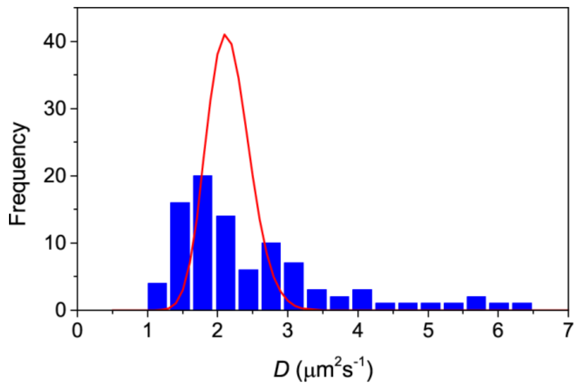

Supplementary Figure 7. Frequency histogram of the diffusion coefficients determined by the SMLT-MSD analyses of 96 trajectories of equal lengths (50 frames) of $ColE_1$ DNA (6 kbp). The red line shows a theoretical statistical probability distribution corresponding to diffusion in a homogeneous environment, with the diffusion coefficient given by mode of the frequency histogram (Eq.S1).

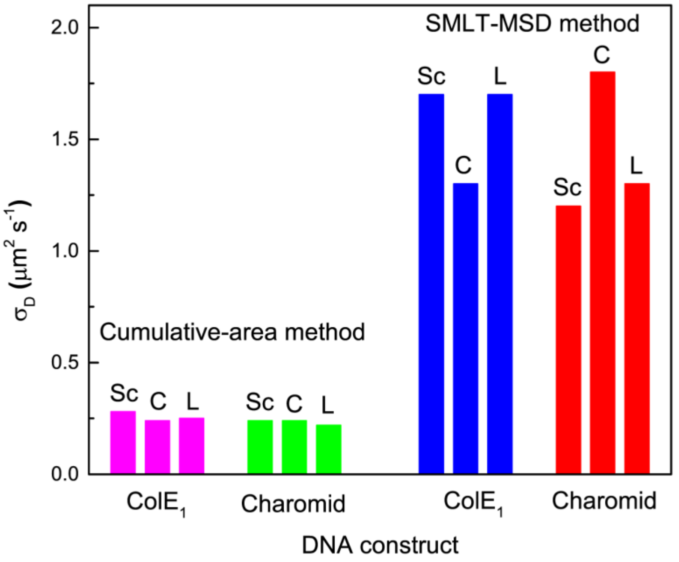

Supplementary Figure 8. Standard deviation of the diffusion coefficients (σ_D) of $ColE_1$ DNA (6 kbp) and Charomid DNA (42 kbp) obtained by the analysis of approximately 100 diffusion trajectories using either the cumulative-area method or SMLT-MSD method. Sc, C, and L denote super-coiled, cyclic, and linear DNA, respectively.

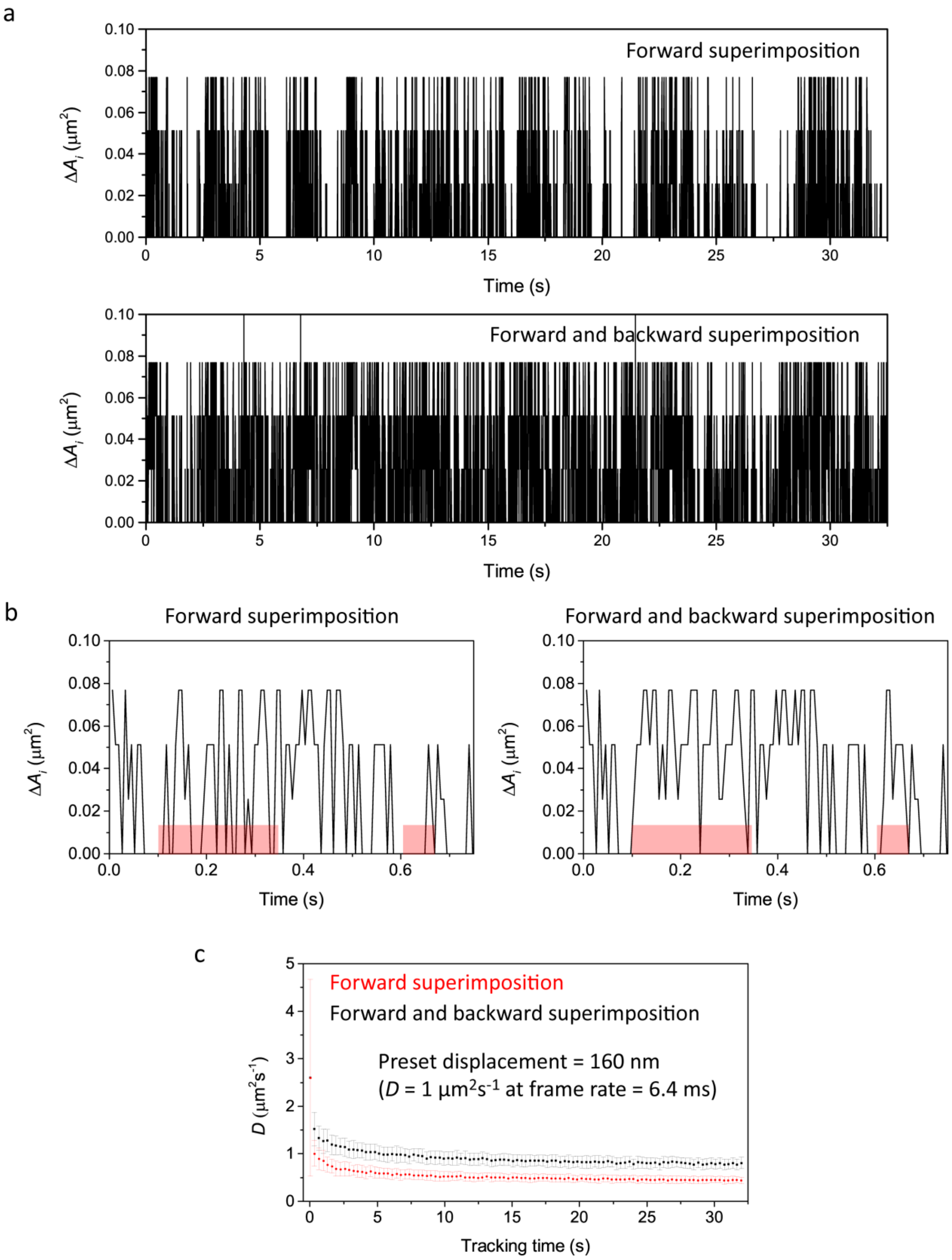
a
Forward superimposition
ΔA_i (μm²)
Time (s)
Forward and backward superimposition
ΔA_i (μm²)
Time (s)
b
Forward superimposition
Forward and backward superimposition
ΔA_i (μm²)
Time (s)
c
Forward superimposition
Forward and backward superimposition
Preset displacement = 160 nm
(D = 1 μm²s⁻¹ at frame rate = 6.4 ms)
D (μm²s⁻¹)
Tracking time (s)

Supplementary Figure 9. Backward superimposition correction of real and simulated single molecule diffusion data. (a) Backward superimposition correction of a single, linear 6 kbp molecule. The cumulative area difference could drop to zero ($\Delta A_i = 0$) because the diffusing molecule occasionally does multiple visits to the same place (see Supplementary Fig. 4). After the backward superimposition, most of the masked area ($\Delta A_i = 0$) in the forward super imposition are uncovered. (b) Magnified figures of the cumulative area difference shown in (a). The cumulative area differences uncovered by the backward superimposition are highlighted in red. (c) Calculated diffusion coefficients of simulated tracks as a function of the tracking time. Before the backward superimposition (red), the diffusion coefficients are underestimated. The gradual decrease in the diffusion coefficient is due to the probabilistic coincidence of the masked area ($\Delta A_i = 0$) in both the forward and backward superimpositions.

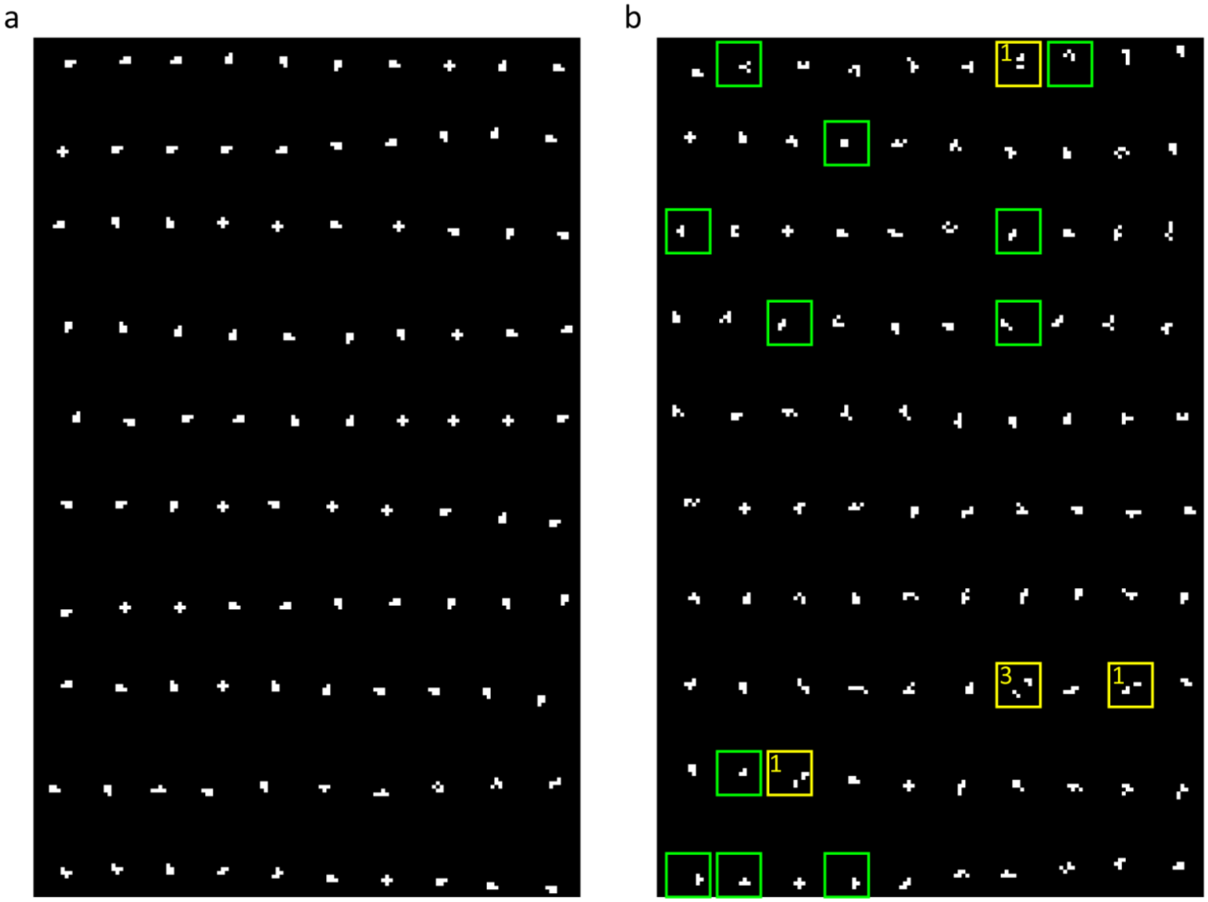

Supplementary Figure 10. Shape fluctuations of the five pixels used to express the area occupied by the molecule in the space. a) shape fluctuations of the five pixels obtained from 100 consecutive frames of nanospheres. B) shape fluctuations of the five pixels obtained from 100 consecutive frames of Charomid 42 kbp. Green squares illustrates occasional appearance of 4 pixels that might arise after identifying the fifth pixel as a noise pixel (Supplementary Fig. 3). This does not affect the calculated diffusion coefficient since 5, 4 and 3 pixels can be used reliably to calculate the diffusion coefficient. Yellow squares illustrates partial splitting of the five pixels. Number indicate the splitting distance (in pixels) in both X and Y directions).

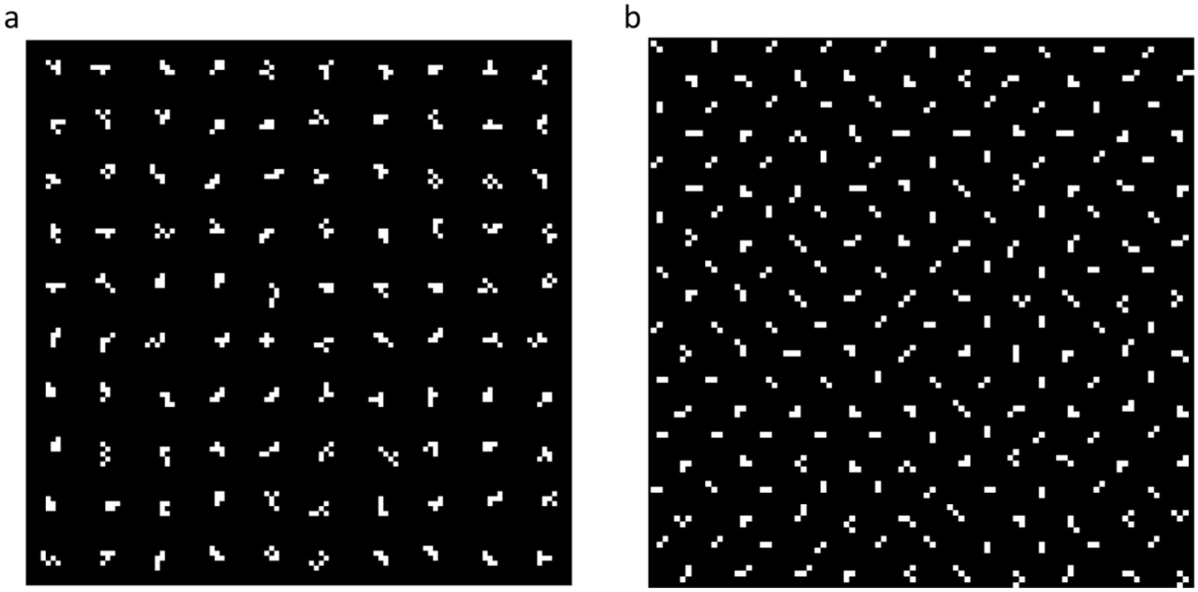

Supplementary Figure 11. Simulation of the effects of both molecular shape and brief defocusing on the shape of the five pixels used to express the area occupied by the molecule in the space. a) Random fluctuations of the shape of five continuous pixels. b) Random fluctuations of the shape of five pixles splitted using 5 pixels in both X and Y directions.

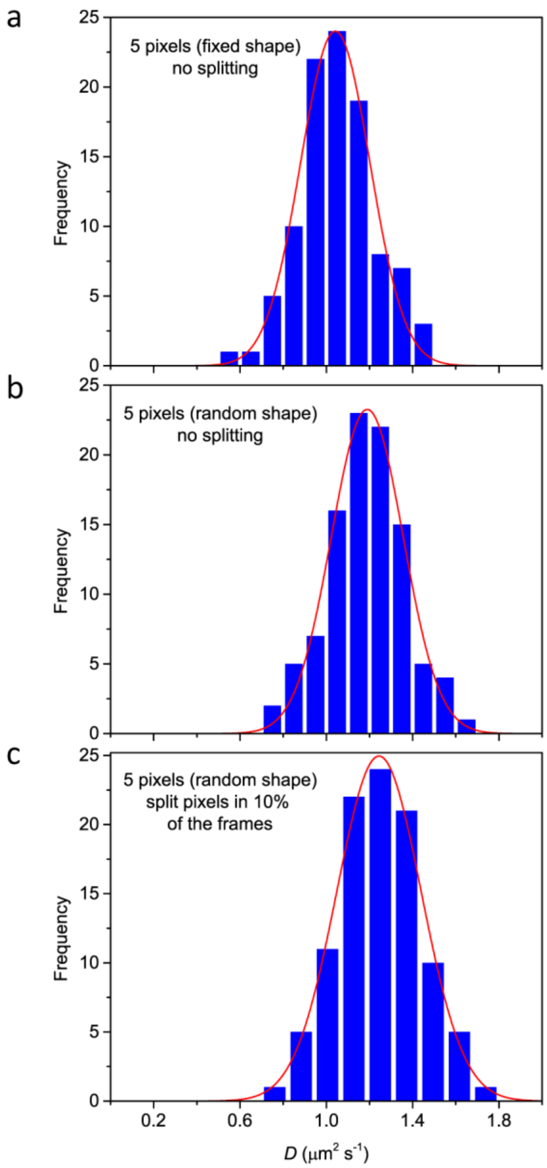

Supplementary Figure 12. Effect of the random shape fluctuations and splitting of the five pixels on the calculated diffusion coefficient using 1000 simulated tracks in two dimensions. 2D trajectories of five pixles diffusing at 1 μm^2 s^{-1} are simulated (500 steps with 0.0064 s time resolutions. (a) Fixed shape of the five pixels is used (Fig. 4b), (b) Random shape of the five pixels is used (Supplementary Fig. 11a), (c) Random shape of the five pixels is used. Pixels are split by 5 pixels in both X and Y directions (Supplementary Fig. 11b).). The diffusion coefficients were calculated using the cumulative-area method and plotted in frequency histograms. The red lines show Gaussian fittings of the histograms.

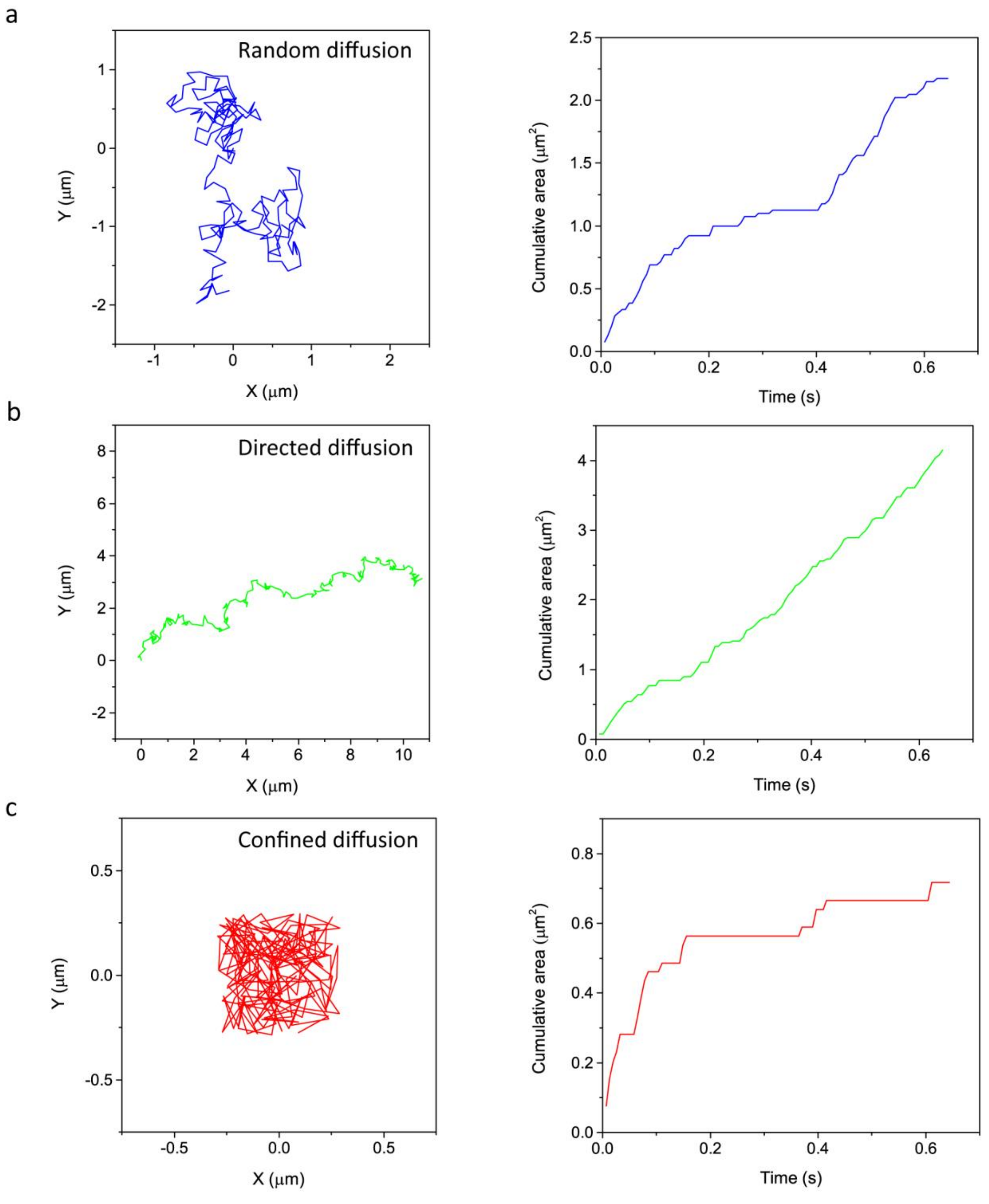
a
Random diffusion
Y (µm)
X (µm)
Cumulative area (µm²)
Time (s)
b
Directed diffusion
Y (µm)
X (µm)
Cumulative area (µm²)
Time (s)
c
Confined diffusion
Y (µm)
X (µm)
Cumulative area (µm²)
Time (s)

Supplementary Figure 13. Simulation of random, directed, and confined motions of single particles in two dimensions. (a) Left: a simulated 2D trajectory of a particle diffusing in a random fashion at *D* = 1.0 $\mu m^2\ s^{-1}$ (frame rate = 6.4ms). Right: cumulative areas at time 0 ~ 0.64 s calculated using the random diffusion trajectory. (b) Left: a simulated 2D trajectory of a particle diffusing in a directed fashion at *D* = 1.0 $\mu m^2\ s^{-1}$ (frame rate = 6.4ms). Right: cumulative areas at time 0 ~ 0.64 s calculated using the directed diffusion trajectory. (c) Left: a simulated 2D trajectory of a particle diffusing in a confined fashion at *D* = 1.0 $\mu m^2\ s^{-1}$ (frame rate = 6.4ms). Right: cumulative areas at time 0 ~ 0.64 s calculated using the confined diffusion trajectory.

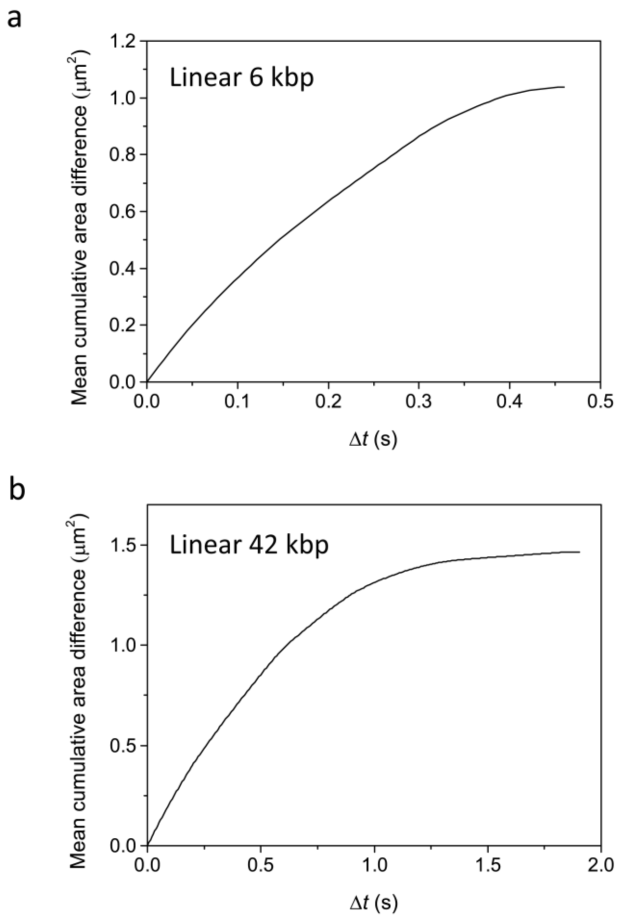

Supplementary Figure 14. Mean cumulative area difference at the time lag Δt of a linear 6 kbp and 42 kbp DNA molecule translating at speed D = 1.3 and 0.98 $\mu m^2\ s^{-1}$, respectively (frame rate = 6.4 ms). The mean cumulative area difference between time frames t_i and t_{i+1} ($1\Delta t$), t_i and t_{i+2} ($2\Delta t$), t_i and t_{i+3} ($3\Delta t$)… are linearly related to Δt at short time lags ($\Delta t < 0.1$ s and $\Delta t < 0.4$ s for the 6 kbp and 42 kbp DNA, respectively). The diffusion coefficients are calculated from the initial slopes of the plots as the statistical error in the calculation of a diffusion coefficient is minimum at the first data points (see Supplementary Fig. 15). At larger time lags, the mean cumulative area difference plot does not contain useful information due to the large statistical errors, similar to the MSD plot (see Supplementary Fig. 15). Diffusion of the DNA molecule in the time scale shorter than its relaxation time is coupled with the conformational states of the molecule. Conversely, diffusion of the molecule in the time

scale longer than the relaxation time is decoupled with the conformational change (i.e. conformational states are averaged within the relaxation time). The mean cumulative area difference obtained for the 42 kbp DNA is proportional to the time lag (Δt) up to Δt = 0.4 s. The relaxation time of the 42kbp DNA (144 ms) is within the linear range of the mean cumulative area difference plot. Therefore, the diffusion coefficient can be accurately determined by the initial slope of the plot (Δt = 6.4 ms), even the time lag is much smaller than the relaxation time.

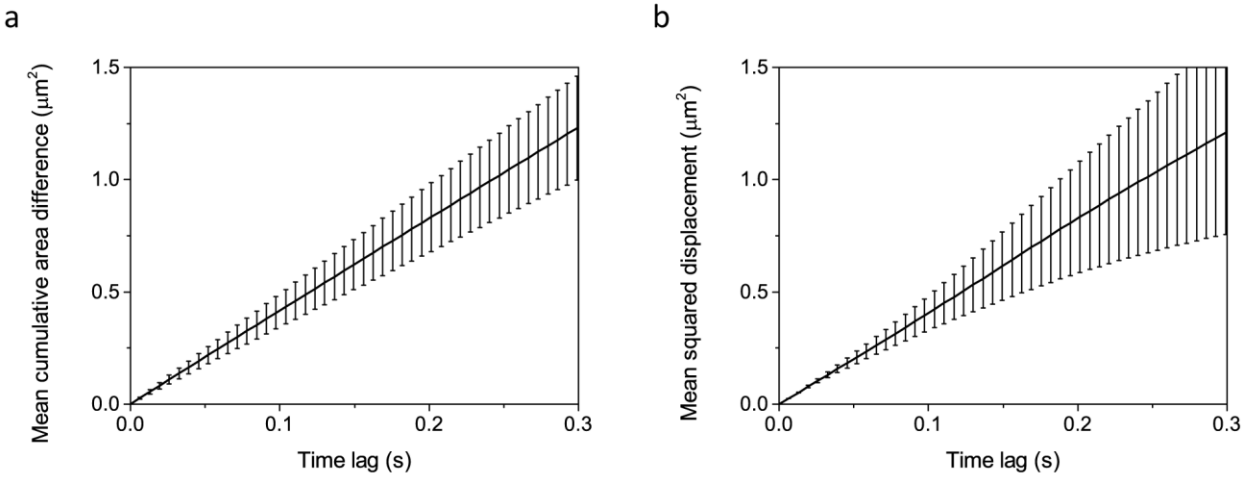

Supplementary Figure 15. Comparison between the (a) mean cumulative area difference and (b) MSD calculated using 100 simulated diffusion trajectories with preset diffusion coefficient of 1 $\mu m^2\ s^{-1}$. Each simulated trajectory contains 500 data points (i.e. trajectory length of 3.2 s at 1Δt = 6.4 ms). The error bars illustrate the standard deviation of the 100 trajectories.

Supplementary Table 1. Time dependent calculations of the diffusion coefficients as a function of the number of pixels and the displacements. Pixel size = 160 nm. Cells, highlighted in yellow, contain the calculated diffusion values that are within ±20% uncertainty (30% of the statistical error, see the main text) of the simulated values (D_p). These results are summarized in Fig. 6.

**Simulation of 6 pix.**		**Total tracking timing (seconds) – Frame rate 6.4ms**														
**d. nm (D_p µm^2 s^{-1})**		0.32	0.64	1.28	1.92	2.56	3.2	3.84	4.48	5.12	5.76	6.4	12.8	19.2	25.6	32
**226 (2)**	**D_μ (µm^2 s^{-1})**	2.23	2.05	1.95	1.87	1.77	1.72	1.69	1.65	1.64	1.62	1.62	1.47	1.41	1.36	1.35
**196 (1.5)**		1.93	1.72	1.6	1.53	1.51	1.44	1.4	1.37	1.32	1.31	1.3	1.23	1.13	1.1	1.08
**160 (1)**		1.56	1.34	1.3	1.2	1.1	1.09	1.07	1.05	1.04	1	0.99	0.92	0.88	0.83	0.78
**113 (0.5)**		1.09	0.95	0.83	0.77	0.73	0.7	0.68	0.68	0.67	0.63	0.63	0.56	0.52	0.49	0.47

**Simulation of 5 pix.**		**Total tracking timing (seconds) – Frame rate 6.4ms**														
**d. nm (D_p µm^2 s^{-1})**		0.32	0.64	1.28	1.92	2.56	3.2	3.84	4.48	5.12	5.76	6.4	12.8	19.2	25.6	32
**226 (2)**	**D_μ (µm^2 s^{-1})**	2.13	1.98	1.84	1.72	1.66	1.63	1.62	1.6	1.57	1.53	1.5	1.4	1.34	1.27	1.23
**196 (1.5)**		1.77	1.65	1.51	1.49	1.43	1.4	1.3	1.3	1.28	1.27	1.25	1.13	1.1	1.07	1.02
**160 (1)**		1.45	1.35	1.2	1.15	1.12	1.05	1.03	1.02	0.98	0.96	0.95	0.9	0.83	0.8	0.8
**113 (0.5)**		1.03	0.9	0.78	0.74	0.77	0.7	0.67	0.67	0.65	0.63	0.6	0.54	0.52	0.5	0.47
**88 (0.3)**		0.88	0.72	0.59	0.55	0.52	0.48	0.47	0.46	0.45	0.45	0.42	0.36	0.35	0.33	0.32
**72 (0.2)**		0.7	0.57	0.47	0.41	0.38	0.37	0.35	0.35	0.33	0.32	0.32	0.28	0.25	0.24	0.23

**Simulation of 4 pix.**		**Total tracking timing (seconds) – Frame rate 6.4ms**														
**d. nm (D_p µm^2 s^{-1})**		0.32	0.64	1.28	1.92	2.56	3.2	3.84	4.48	5.12	5.76	6.4	12.8	19.2	25.6	32
**226 (2)**	**D_μ (µm^2 s^{-1})**	1.98	1.79	1.7	1.64	1.6	1.5	1.47	1.48	1.45	1.44	1.42	1.29	1.23	1.22	1.22
**196 (1.5)**		1.74	1.54	1.4	1.37	1.37	1.34	1.3	1.26	1.22	1.2	1.2	1.1	1.1	1	0.99
**160 (1)**		1.33	1.25	1.12	1.09	1.06	1.01	0.98	0.95	0.93	0.92	0.9	0.85	0.8	0.76	0.76
**113 (0.5)**		0.92	0.83	0.76	0.68	0.66	0.64	0.63	0.6	0.59	0.58	0.57	0.53	0.49	0.46	0.44
**88 (0.3)**		0.8	0.68	0.55	0.54	0.5	0.48	0.45	0.43	0.41	0.42	0.42	0.36	0.32	0.31	0.31
**72 (0.2)**		0.65	0.52	0.45	0.41	0.37	0.35	0.35	0.33	0.3	0.3	0.3	0.26	0.25	0.23	0.22

**Simulation of 3 pix.**		**Total tracking timing (seconds) – Frame rate 6.4ms**														
**d. nm (D_p µm^2 s^{-1})**		0.32	0.64	1.28	1.92	2.56	3.2	3.84	4.48	5.12	5.76	6.4	12.8	19.2	25.6	32
**226 (2)**	**D_μ (µm^2 s^{-1})**	1.72	1.64	1.51	1.44	1.4	1.39	1.36	1.33	1.31	1.27	1.3	1.21	1.17	1.14	1.14
**196 (1.5)**		1.4	1.37	1.26	1.25	1.2	1.17	1.14	1.1	1.1	1.08	1.07	1.03	0.97	0.93	0.9
**160 (1)**		1.2	1.1	1.05	0.98	0.95	0.93	0.89	0.88	0.86	0.85	0.83	0.77	0.75	0.7	0.68
**113 (0.5)**		0.85	0.76	0.66	0.65	0.61	0.61	0.56	0.55	0.54	0.53	0.52	0.5	0.43	0.44	0.42
**88 (0.3)**		0.67	0.6	0.5	0.47	0.43	0.42	0.4	0.4	0.37	0.37	0.35	0.33	0.31	0.29	0.28
**72 (0.2)**		0.53	0.46	0.39	0.37	0.33	0.32	0.3	0.3	0.28	0.28	0.27	0.25	0.22	0.21	0.21

Supplementary Table 2. Time dependent calculations of the diffusion coefficients as a function of the number of pixels and the displacements. Pixel size = 100 nm. Cells, highlighted in yellow, contain the calculated diffusion values that are within ±20% uncertainty (30% of the statistical error, see the main text) of the simulated values (D_p).

Simulation of 5 pix.		Total tracking timing (seconds) – Frame rate 6.4ms														
d. nm (D_p μm^2 s^{-1})		0.32	0.64	1.28	1.92	2.56	3.2	3.84	4.48	5.12	5.76	6.4	12.8	19.2	25.6	32
226 (2)	D_μ (μm^2 s^{-1})	1.30	1.24	1.13	1.13	1.08	1.07	1.05	1.04	1.04	1.03	1.01	0.97	0.93	0.91	0.91
196 (1.5)		1.13	1.07	1.01	0.96	0.95	0.93	0.89	0.89	0.89	0.89	0.87	0.81	0.79	0.78	0.76
160 (1)		0.95	0.87	0.82	0.77	0.74	0.72	0.73	0.71	0.70	0.68	0.67	0.65	0.61	0.60	0.59
113 (0.5)		0.68	0.62	0.55	0.52	0.50	0.47	0.47	0.46	0.44	0.44	0.44	0.40	0.38	0.37	0.36
88 (0.3)		0.56	0.47	0.43	0.38	0.36	0.36	0.35	0.34	0.33	0.32	0.31	0.28	0.27	0.27	0.25
72 (0.2)		0.45	0.39	0.32	0.30	0.28	0.28	0.26	0.26	0.26	0.25	0.24	0.21	0.20	0.19	0.19
51 (0.1)		0.31	0.25	0.22	0.2	0.18	0.18	0.17	0.16	0.16	0.16	0.15	0.13	0.12	0.12	0.1

Simulation of 4 pix.		Total tracking timing (seconds) – Frame rate 6.4ms														
d. nm (D_p μm^2 s^{-1})		0.32	0.64	1.28	1.92	2.56	3.2	3.84	4.48	5.12	5.76	6.4	12.8	19.2	25.6	32
226 (2)	D_μ (μm^2 s^{-1})	1.10	1.06	1.01	0.98	0.97	0.95	0.94	0.94	0.91	0.92	0.91	0.87	0.85	0.84	0.82
196 (1.5)		1.03	0.97	0.89	0.87	0.86	0.84	0.83	0.83	0.82	0.81	0.80	0.76	0.73	0.73	0.70
160 (1)		0.85	0.78	0.77	0.72	0.70	0.69	0.65	0.67	0.66	0.64	0.65	0.60	0.58	0.57	0.55
113 (0.5)		0.64	0.60	0.53	0.49	0.48	0.47	0.45	0.44	0.44	0.43	0.41	0.38	0.37	0.37	0.35
88 (0.3)		0.50	0.44	0.38	0.37	0.35	0.33	0.32	0.32	0.32	0.31	0.30	0.27	0.26	0.25	0.24
72 (0.2)		0.43	0.35	0.30	0.28	0.27	0.27	0.26	0.25	0.24	0.23	0.24	0.20	0.19	0.19	0.18
51 (0.1)		0.3	0.24	0.2	0.18	0.17	0.16	0.16	0.15	0.15	0.15	0.14	0.12	0.11	0.11	0.1

Simulation of 3 pix.		Total tracking timing (seconds) – Frame rate 6.4ms														
d. nm (D_p μm^2 s^{-1})		0.32	0.64	1.28	1.92	2.56	3.2	3.84	4.48	5.12	5.76	6.4	12.8	19.2	25.6	32
226 (2)	D_μ (μm^2 s^{-1})	0.91	0.88	0.85	0.82	0.82	0.81	0.79	0.78	0.79	0.78	0.79	0.75	0.73	0.72	0.70
196 (1.5)		0.85	0.81	0.77	0.75	0.75	0.73	0.72	0.72	0.72	0.70	0.70	0.66	0.64	0.64	0.62
160 (1)		0.74	0.70	0.66	0.63	0.62	0.61	0.59	0.59	0.58	0.57	0.57	0.54	0.52	0.52	0.51
113 (0.5)		0.56	0.52	0.46	0.44	0.43	0.41	0.41	0.41	0.40	0.39	0.39	0.35	0.34	0.34	0.33
88 (0.3)		0.45	0.40	0.34	0.34	0.32	0.31	0.29	0.28	0.29	0.28	0.28	0.26	0.24	0.24	0.22
72 (0.2)		0.36	0.33	0.27	0.27	0.25	0.24	0.23	0.23	0.22	0.22	0.21	0.20	0.18	0.17	0.17
51 (0.1)		0.24	0.2	0.18	0.16	0.16	0.15	0.15	0.14	0.14	0.13	0.12	0.12	0.1	0.1	0.1

Supplementary Table 3. Ranges of the tracking times for different diffusion coefficients based on the experimental data

D μm^2 s^{-1} (simulated)	Tracking time (s) *	Reference sample	*D* μm^2 s^{-1} (reference sample)
2	0.32 – 1.92	Supercoiled ColE1 (6 kbp)	1.8
1.5	0.32 – 1.92	Linear ColE1 (6 kbp)	1.6
1	1.28 – 6.4	Linear Charomid (42 kbp)	0.99
0.5	5.12 – 12.8	Lambda DNA	0.6 (data not shown)
0.3	6.4 – 32	Extrapolated	---

* as referred by the bars in Fig. 6.
